# Parallel Metabolomics and Lipidomics of a PSMA/GCPII Deficient Mouse Model Reveal Alteration of NAAG Levels and Brain Lipid Composition

**DOI:** 10.1021/acschemneuro.3c00494

**Published:** 2024-02-20

**Authors:** František Sedlák, Aleš Kvasnička, Barbora Marešová, Radana Brumarová, Dana Dobešová, Kateřina Dostálová, Karolína Šrámková, Martin Pehr, Pavel Šácha, David Friedecký, Jan Konvalinka

**Affiliations:** † Institute of Organic Chemistry and Biochemistry, Czech Academy of Sciences, Prague 6 166 10, Czechia; ‡ Institute of Biochemistry and Experimental Oncology, First Faculty of Medicine, Charles University, Prague 2 110 01, Czechia; § First Department of Internal Medicine - Hematology, Charles University General Hospital in Prague, Prague 110 01, Czechia; ∥ Laboratory for Inherited Metabolic Disorders, Department of Clinical Biochemistry, University Hospital Olomouc, and Faculty of Medicine and Dentistry, Palacký University Olomouc, Zdravotníku° 248/7, Olomouc 779 00, Czechia; ⊥ Third Department of Medicine − Department of Endocrinology and Metabolism of the first Faculty of Medicine and General University Hospital in Prague, Charles University, Prague 110 01, Czechia; # Department of Biochemistry, Faculty of Science, Charles University, Hlavova 8, Prague 128 00, Czechia

**Keywords:** lipidomics, metabolomics, *N*-acetyl-aspartyl-glutamate, glutamate carboxypeptidase II, FOLH1, folyl-poly-γ-glutamyl hydrolase I

## Abstract

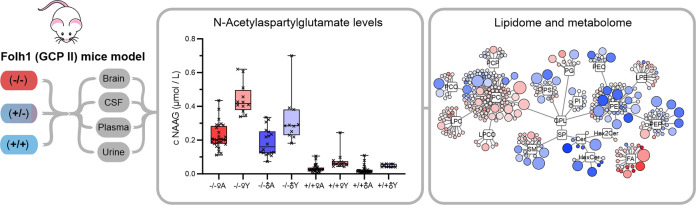

Glutamate carboxypeptidase II (GCPII, also known as PSMA or FOLH1) is responsible for the cleavage of *N*-acetyl-aspartyl-glutamate (NAAG) to *N*-acetyl-aspartate and glutamate in the central nervous system and facilitates the intestinal absorption of folate by processing dietary folyl-poly-γ-glutamate in the small intestine. The physiological function of GCPII in other organs like kidneys is still not known. GCPII inhibitors are neuroprotective in various conditions (e.g., ischemic brain injury) *in vivo*; however, their utilization as potential drug candidates has not been investigated in regard to not yet known GCPII activities. To explore the GCPII role and possible side effects of GCPII inhibitors, we performed parallel metabolomic and lipidomic analysis of the cerebrospinal fluid (CSF), urine, plasma, and brain tissue of mice with varying degrees of GCPII deficiency (fully deficient in *Folh1*, −/–; one allele deficient in *Folh1*, +/–; and wild type, +/+). Multivariate analysis of metabolites showed no significant differences between wild-type and GCPII-deficient mice (except for NAAG), although changes were observed between the sex and age. NAAG levels were statistically significantly increased in the CSF, urine, and plasma of GCPII-deficient mice. However, no difference in NAAG concentrations was found in the whole brain lysate likely because GCPII, as an extracellular enzyme, can affect only extracellular and not intracellular NAAG concentrations. Regarding the lipidome, the most pronounced genotype-linked changes were found in the brain tissue. In brains of GCPII-deficient mice, we observed statistically significant enrichment in phosphatidylcholine-based lipids and reduction of sphingolipids and phosphatidylethanolamine plasmalogens. We hypothesize that the alteration of the NAA-NAAG axis by absent GCPII activity affected myelin composition. In summary, the absence of GCPII and thus similarly its inhibition do not have detrimental effects on metabolism, with just minor changes in the brain lipidome.

## 1 Introduction

Glutamate carboxypeptidase II (GCPII, EC 3.4.17.21), also known as prostate-specific membrane antigen (PSMA), folyl-poly-γ-glutamyl hydrolase I (FOLH1), or *N*-acetylated-alpha-linked acidic dipeptidase I (NAALadase I), is a transmembrane zinc exopeptidase with two known basic physiological functions: degradation of the peptide neurotransmitter *N*-acetyl-aspartyl-glutamate (NAAG) to *N*-acetyl-aspartyl-aspartate (NAA) and glutamate in the nervous system^1^ and processing of dietary folyl-poly-γ-glutamates to facilitate folate absorption in the intestine.^2^ The closest homologue of GCPII is glutamate carboxypeptidase III (GCPIII, EC:3.4.17.21).^3^ In addition to GCPII substrates, GCPIII can efficiently cleave β-citryl-glutamate (BCG).^4,5^

**Figure 1:**
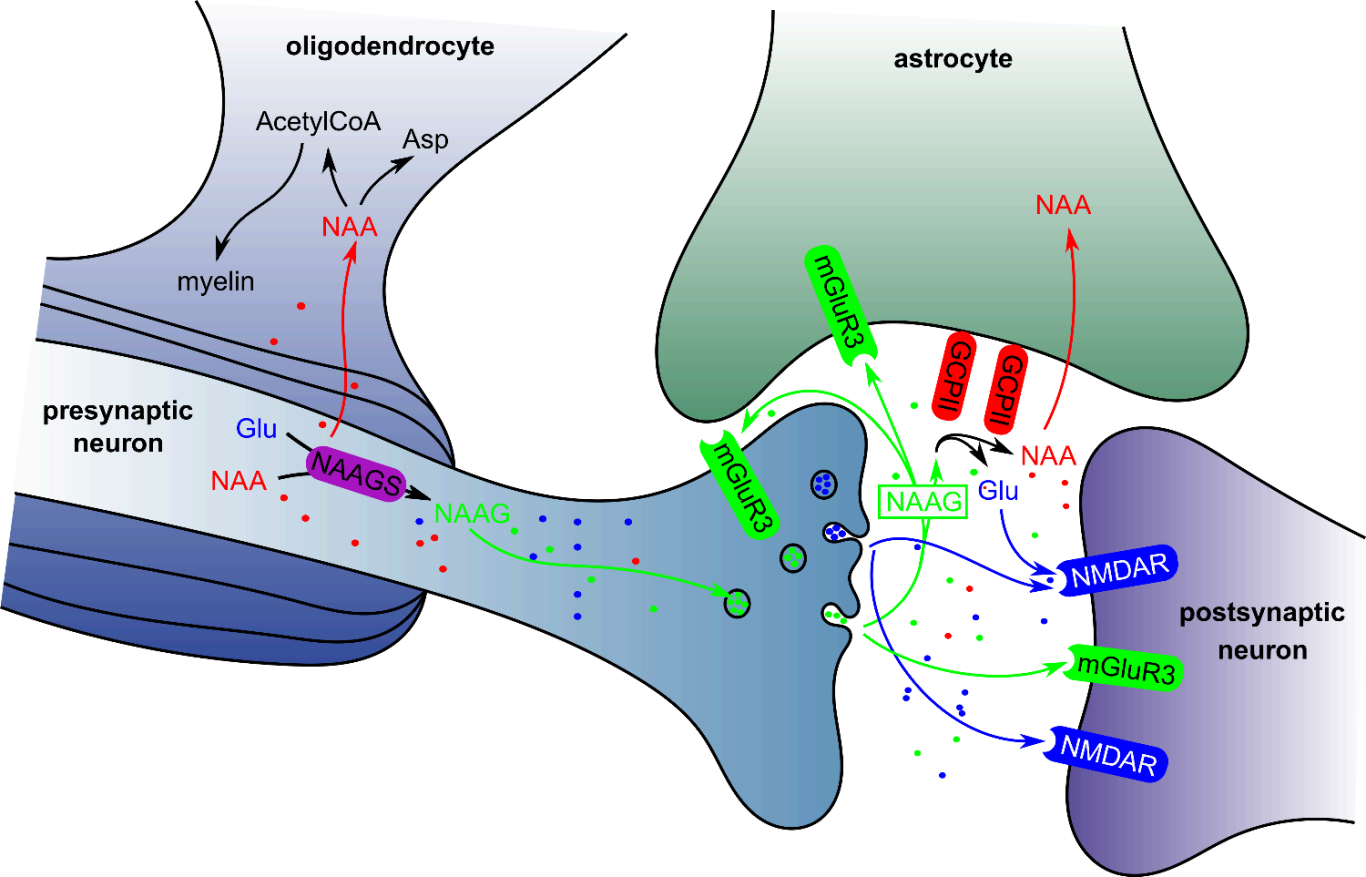
Model of NAAG metabolism in the central nervous system. To simplify the figure, not all of the cofactors are shown for enzymatic reactions. The mechanism is described in detail in the main text. The abbreviations correspond to NAA, *N*-acetyl-aspartyl-aspartate; NAAGS, *N*-acetylaspartylglutamate synthetase; NAAG, *N*-acetyl-aspartyl-glutamate; Asp, aspartate; Glu, glutamate; mGluR3, metabotropic glutamate receptors; and NMDAR, *N*-methyl-d-aspartate receptor.

NAAG is produced by *N*-acetylaspartylglutamate synthetase (NAAGS, EC 6.3.2.41) in neurons and transported to synaptic vesicles (Figure 1). Upon depolarization of the neuron, NAAG, along with other neurotransmitters, is released into the synaptic cleft, where it activates metabotropic glutamate receptors (mGluR3) on both neurons and astrocytes.^6,7^ Here, NAAG reduces the level of excessive neurotransmission and thus protects neurons. Presynaptically, NAAG decreases calcium intake during depolarization, therefore acting as negative feedback in neurotransmission.^8^ Postsynaptically, NAAG increases the expression of receptors of inhibitory neurotransmitters.^9^ Astrocytes are stimulated by NAAG to produce neuroprotective TGF-β.^10^

Because NAAG is rapidly removed by GCPII activity, the indirect increase in NAAG levels due to GCPII inhibition also shows a neuroprotective effect. Treatment by GCPII inhibitors has been effective in animal models of ischemic brain injury,^11−13^ traumatic brain injury,^14^ amyotrophic lateral sclerosis,^15^ inflammatory/neuropathic pain,^16−19^ and schizophrenia.^20,21^

The metabolic effects of GCPII deficiency, whether in the brain or elsewhere in the organism, have not been thoroughly studied. Regarding the not yet known physiological role of GCPII in organs such as kidneys and prostate, it is important to evaluate the metabolic consequences of GCPII absence. Because GCPII-deficient mice can model the action of GCPII inhibitors *in vivo*, it can be valuable to predict possible side effects of GCPII inhibitors as drug candidates. Moreover, utilizing a GCPII-deficient mouse could be helpful for following long-term effects that will be challenging to analyze by application of GCPII inhibitors.

Several research groups have attempted to generate GCPII-deficient mice; however, the resulting phenotypes of these mice are quite controversial. Although embryonic lethality was observed by some,^22,23^ there were no significant harmful effects nor embryonic development alterations found by us and others.^24−26^ A number of studies have even demonstrated a neuroprotective effect of GCPII deletion.^26,27^ The only non-neuronal phenotype observed in GCPII-deficient mice was enlargement of seminal vesicles of aged males.^24^

It has not yet been studied how the absence of GCPII affects metabolism in the central and peripheral nervous system, but we have evidence of the association of increased NAAG concentrations in CSF with impaired myelination^28^ and linkage of several white matter diseases and leukodystrophies with alteration of NAAG levels in CSF.^29,30^ A possible explanation could be that NAA serves as a source of acetyl groups for myelin synthesis in oligodendrocytes.^28,31,32^ Also, deficiency of *N*-acetyltransferase-8-like enzyme (NAT8L) responsible for the production of NAA in neurons results in a decrease in sphingomyelin and sulfatide levels in mouse brains.^33^ Nevertheless, the direct causative link between the NAAG concentration and brain lipid content remains unproven.

**Figure 2:**
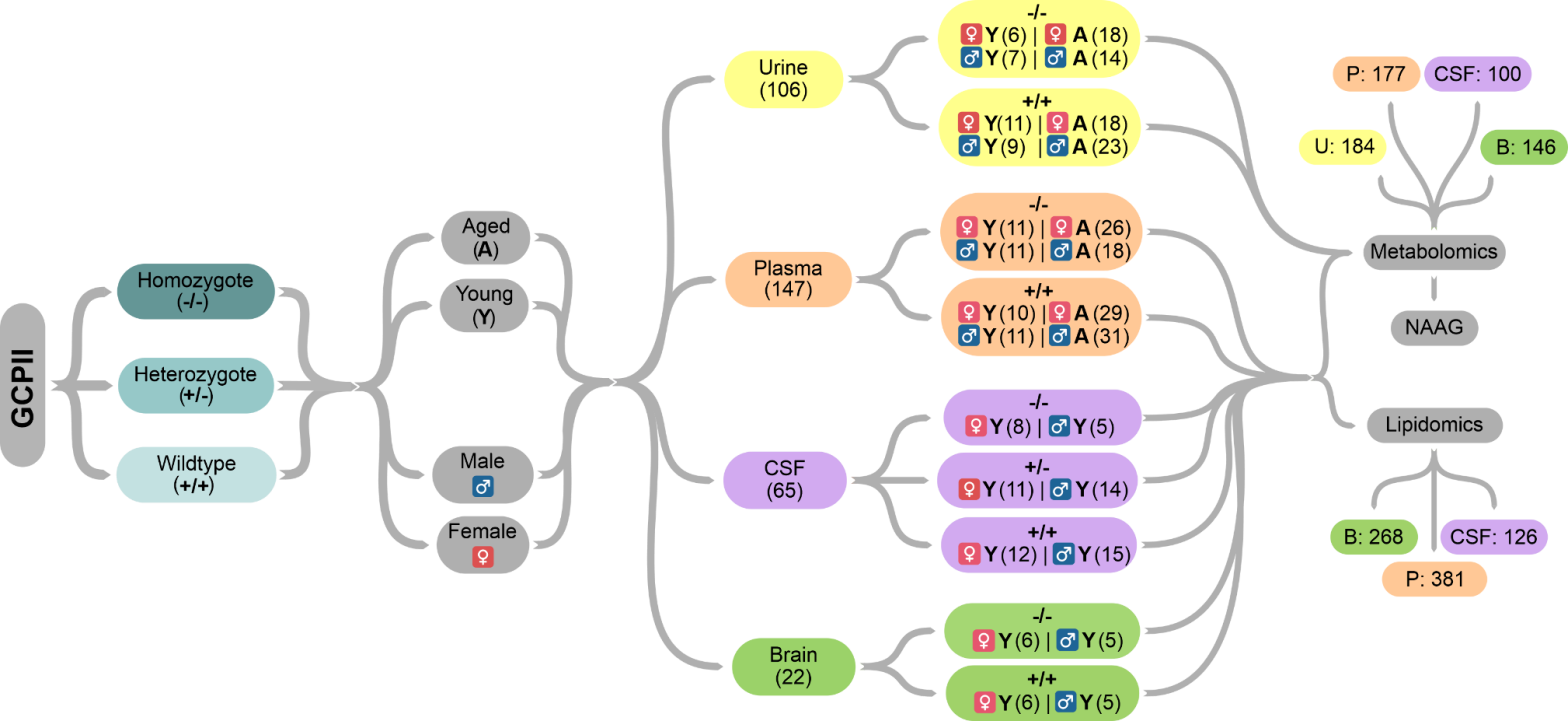
Design of the experiment. The number of biological replicates of mice used for each group divided by GCPII genotype (fully deficient in *Folh1*: −/–, one allele deficient in *Folh1*: +/–, and wild type: +/+), sex (males: ♂, females: ♀), and age (young, below 25 weeks: Y; aged, over 60 weeks: A) are shown in parentheses. The number of metabolites and lipids identified in each biological material analyzed (urine: U, plasma: P, cerebrospinal fluid: CSF, and brain: B) is color-coded by material.

It is well-known that multiomics approaches are powerful tools in the study of metabolism.^34^ For this purpose, we applied comprehensive targeted metabolomics and lipidomics approaches to study the brain tissue and biological fluid of GCPII-deficient mice and their wild-type counterparts (Figure 2).

## 2 Results

Comprehensive metabolomic and lipidomic analysis of urine, plasma, CSF, and brain tissue was performed to describe biochemical changes. The results of univariate and multivariate statistical analyses are summarized in the following sections below. However, it should be noted that the significance of univariate statistical methods is affected by the number of observations (samples) in the studied groups.^35^ In our case, we were limited by the lower number of samples of brain tissue and CSF compared to plasma and urine due to the difficulty of the collection technique (Figure 2). Therefore, it must be considered that the statistical significance cannot be completely compared between studied materials. It agrees with the a priori calculation of sample size and statistically significant effect size for *t* test, where for the smallest and largest groups of 6/5 and 26/29 samples, the Cohens’ *d* was calculated as 1.9 and 0.8, respectively.

### 2.1 Targeted Metabolomic Analysis

A total of 251 unique metabolites were identified in this study, specifically 184 metabolites in urine, 177 in plasma, 100 in CSF, and 146 in the brain tissue, respectively (Table 1).

**Table 1:** Detailed Distribution of Metabolites Identified in the Studied Materials with Classification into Groups According to Their Biochemical Relation

metabolite group	number of metabolites			
	urine	plasma	CSF	brain
amines	10	10	9	11
proteinogenic amino acids	19	20	19	18
conjugates of amino acids	30	35	11	16
dipeptides	6	4	3	3
coenzymes & vitamins	9	9	6	7
hydroxyl chain acylcarnitines	5	11	1	7
long/very long chain acylcarnitines	7	16	1	12
medium chain acylcarnitines	8	7	0	5
short chain acylcarnitines	6	7	5	6
organic acids	33	23	8	13
phosphosaccharides	4	0	4	9
purine/pyrimidine bases and ribosides	28	21	19	15
purine/pyrimidine conjugates	1	0	0	4
purine/pyrimidine nucleotides	2	0	3	11
saccharides	16	14	11	9
total	**184**	**177**	**100**	**146**

**Figure 3:**
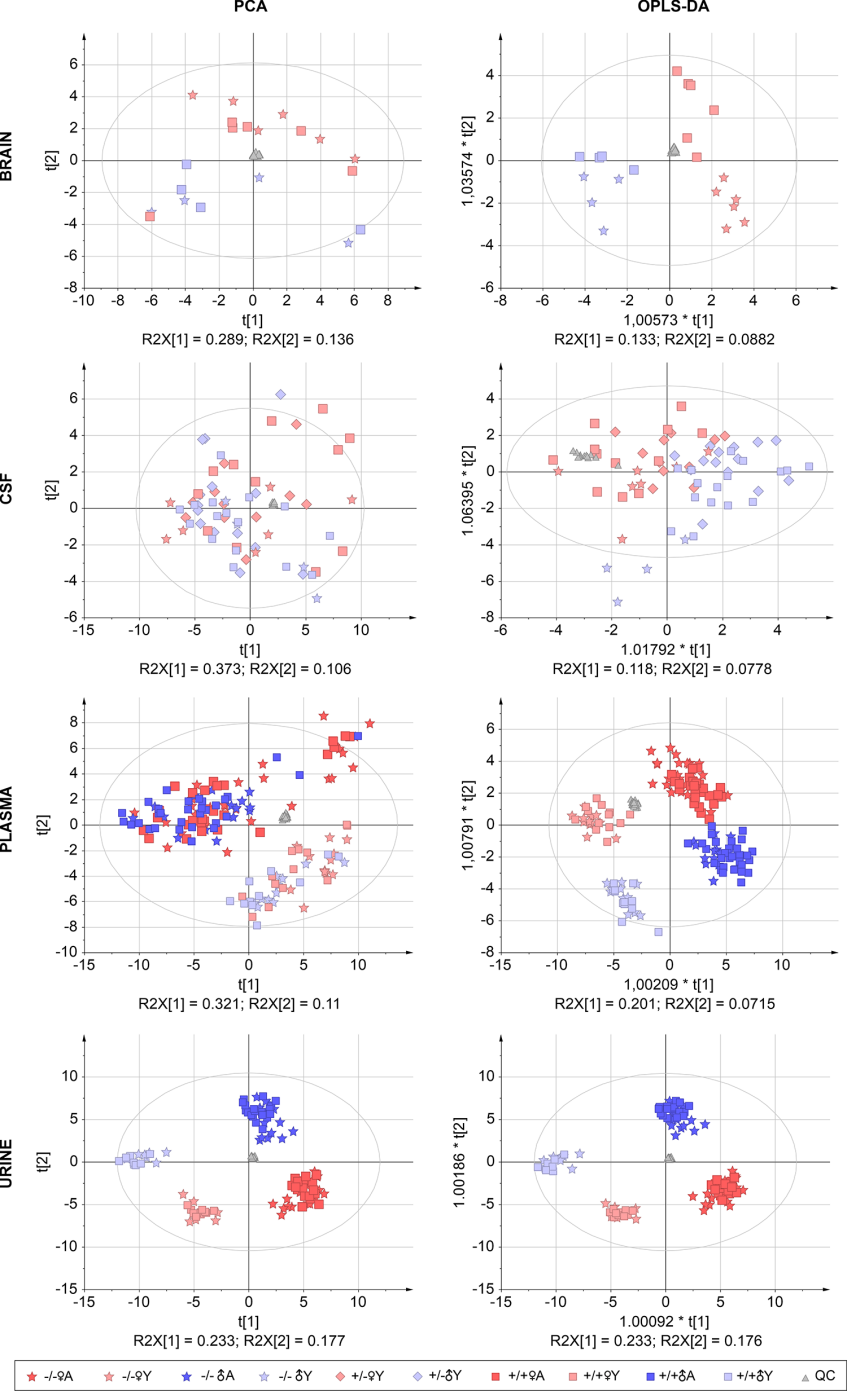
Multivariate analysis (unsupervised PCA, left; supervised OPLS-DA, right) of metabolite profiles in the brain, CSF, plasma, and urine samples of mice with different *Folh1* genotypes, sex, and age. The number of biological replicates is described in detail in the Figure 2. Source data are provided in Table S1 and normality testing in Table S2.

To overview the changes in metabolome, multivariate analysis was applied (Figure 3). NAAG was excluded from the targeted metabolomic analysis data processing and was evaluated separately (further explained in Section 3.3). In the urine samples, unsupervised principal component analysis (PCA) and supervised orthogonal partial least-squares discriminant analysis (OPLS-DA) showed strict separation of the groups based on age and sex of the studied mice. Only a slight discrimination between the *Folh1* −/– and *Folh1* +/+ mice was seen in both ♀A and ♂A in the urine samples. A similar trend of discrimination was seen in the OPLS-DA of the plasma samples, whereas only separation by age was observed in PCA. The CSF and brain tissue lysates have been studied in only young mice. In the brain tissue lysate, separation by sex could be observed in OPLS-DA and in PCA. In CSF, the separation was observed only in OPLS-DA. However, because of the difficulty of collection and the resulting smaller number of samples per group, the analysis is influenced by lower statistical significance.

After the Bonferroni multiple testing correction (BF), most of the metabolites did not show statistically significant changes in the univariate analysis (Table S3). Only a few metabolites were assessed as statistically significant. Trends of significant metabolites (and some of those just below the adjusted *p* value threshold) showed, for example, decreased levels of several saccharides (glucose, arabitol, myoinositol, glucuronic acid) in the urine (with *p* values 9.6 × 10^–3^, 5.8 × 10^–3^, and 3.8 × 10^–2^, 7.5 × 10^–1^, respectively) and similarly in the CSF (with *p* values 9.1 × 10^–4^, 1.9 × 10^–1^, 5.3 × 10^–3^, and 1.4 × 10^–2^, respectively) of −/–♂Y compared to +/+♂Y. Alterations in the purine metabolism (adenosine, hypoxanthine, 1-methylxanthine) were observed in the plasma (with *p* values 1.1 × 10^–4^, 5.8 × 10^–2^, and not measured, respectively) and urine (with *p* values 2.6 × 10^–1^, 2.1 × 10^–2^, and 1.9 × 10^–4^, respectively) samples from the −/–♀A group compared to +/+♀A. An isolated finding of increased acylglycines (isobutyrylglycine, methylbutyrylglycine, hexanoylglycine) in the plasma (with *p* values 8.8 × 10^–4^, 7.6 × 10^–4^, and 3.85.4 × 10^–4^, respectively) of −/–♀Y compared to +/+♀Y was also found. Similarly, in the brain tissue, only a decrease of phosphocreatine (*p* value = 2.5 × 10^–4^) in the −/–♀Y compared to +/+♀Y mice and an increase of asparagine (*p* value = 8.0 × 10^–4^) in the −/–♂Y compared to +/+♂Y mice were observed. In summary, we found no clear systematic differentiation by GCPII genotypes in multivariate and univariate analyses of metabolome patterns of CSF, urine, blood plasma, and brain tissue.

### 2.2 Targeted Lipidomic Analysis

Overall, 518 unique lipids were identified, of which 130 were fully annotated by both acyl chains, not considering lipids with single acyl chain, e.g., lyso-lipids. The exact number of lipids identified in each material is as follows: 126 lipids in the CSF, 268 in the brain tissue, and 381 in the plasma. The identified lipids belonged to 17 lipid classes and subclasses specified in Table 2. Confirmation of the correct identification of lipids was performed using lipid elution patterns (Figure S1).

**Table 2:** Number of Lipids Identified in the Studied Materials with Classification into Classes

lipid class	abbreviation	number of lipids		
		plasma	CSF	brain
cholesteryl esters	CE	0	4	0
ceramides	Cer	4	4	6
free fatty acids	FA	21	21	22
dihexosylceramides	Hex2Cer	0	0	1
monohexosylceramides	HexCer	2	2	6
lysophosphatidylcholine	LPC	32	9	21
lysophosphatidylcholine plasmanyls	LPCO	6	0	5
lysophosphatidylethanolamines	LPE	14	7	15
lysophosphatidylethanolamine plasmanyls	LPEO	0	0	1
phosphatidylcholines	PC	123	30	64
phosphatidylcholine plasmanyls/plasmalogens	PCO/PCP	71	15	22
phosphatidylethanolamines	PE	28	12	27
phosphatidylethanolamine plasmanyls/plasmalogens	PEO/PEP	20	5	40
phosphatidylglycerols	PG	0	0	2
phosphatidylinositols	PI	14	0	4
phosphatidylserines	PS	4	0	10
sphingomyelins	SM	42	17	22
total		**381**	**126**	**268**

**Figure 4:**
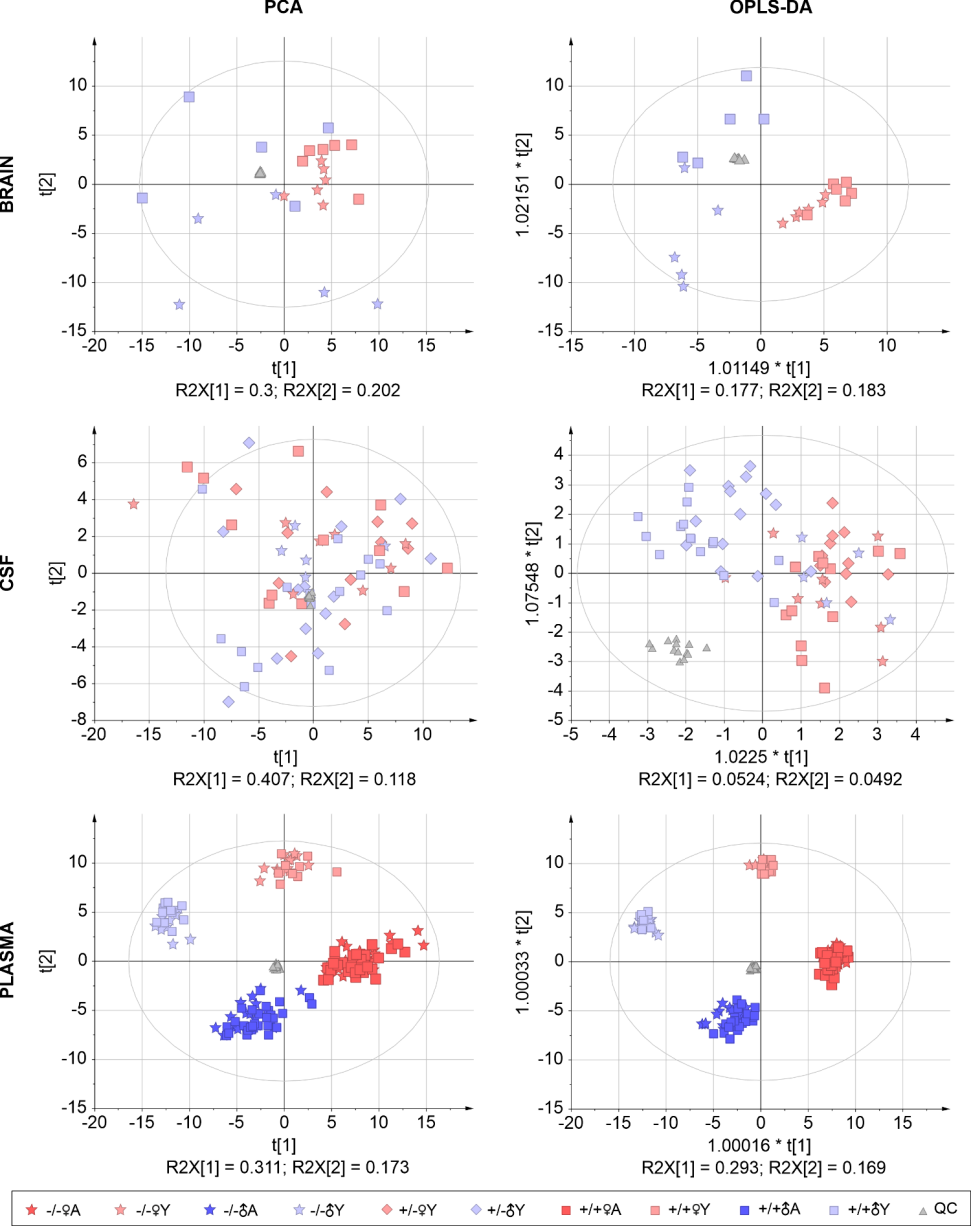
Multivariate analysis (unsupervised PCA, left; supervised OPLS-DA, right) of lipid profiles in the brain, CSF, and plasma samples from mice of different *Folh1* genotypes, sex, and age. The number of biological replicates is described in detail in the Figure 2. Source data are provided in Table S1 and normality testing in Table S2.

The changes in the lipidome across all the studied biological materials were first evaluated by multivariate statistical approaches (Figure 4). In the plasma samples, both PCA and OPLS-DA clustered data into four groups based on age and sex but not based on genetic differences. In the CSF, there was no separation occurring in the PCA plot, but separation was observed based on sex in the OPLS-DA plot. Closer examination of the CSF’s OPLS-DA plot revealed separation of the −/–♂Y group from the +/–♂Y and +/+♂Y groups. Interestingly, this trend did not occur in the female group. The most pronounced separation based on genetic characteristics was observed in the brain. There was a clear separation between the −/−/♂Y and +/+♂Y groups in both OPLS-DA and PCA.

**Figure 5:**
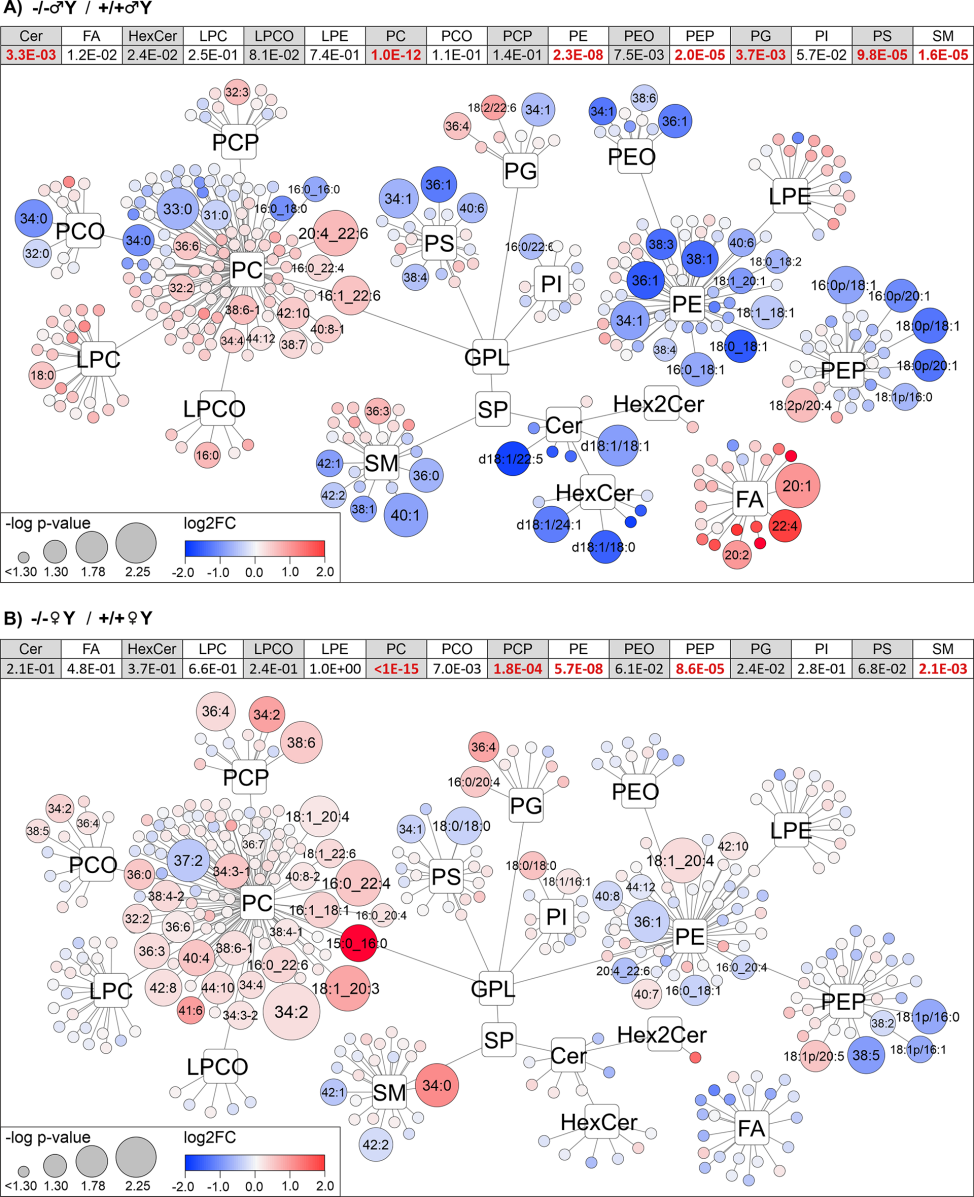
Overview of changes in brain lipidome of males (A) and females (B) across all lipid classes from *Folh1* +/+ and *Folh1* −/– mice. The color of nodes corresponds to log2FC, and the size of the nodes corresponds to −log of the *p* value. Lipids with a log *p* value > 1.3 are shown with labels. Values in the table above the figure correspond to a cumulative *p* value for each lipid class calculated via Fisher’s method (values in red bold are significant after BF, detail in Table S3). Labels are shown based on the level of identification confidence as a sum or acyl-chain-specific formula. The numbers in parentheses after the group type correspond to the numbers of biological replicates. Results are also provided for brain tissue and CSF comparing females versus males (Figure S2) and also for CSF of males and females across all lipid classes comparing *Folh1*+/+ and *Folh1* −/– mice (Figure S3).

Given that separation for genetic variants was the most pronounced in the brain and CSF, the focus was shifted to the investigation of specific changes in the lipidome. Because the sexual dimorphism in the brain metabolome was described previously,^36^ we decided to analyze both sexes separately. Indeed, we confirmed the brain and even CSF lipidome dependency on the sex (+/+♂Y versus +/+♀Y) as can be seen on the visualization of lipidome trends in Cytoscape (Figure 5 and Figure S2). For example, differences were previously observed in higher total levels of fatty acids 16:0 and 18:0 in males and, conversely, elevated levels of 22:6, 20:4, or 22:4 in females.^36^ Similarly, several alterations in the composition of the acyl chains between the male and female group can be observed in our data, especially in the PC, PE, PEP, and LPC species, where the difference was mainly observed in the aforementioned 16:0, 18:0, 20:4, and 22:4 acyl chain lipid species in the brain and CSF (Figure S2).

After applying BF, there was no significant change in any of the lipids in the brain or CSF, indicating less intense alteration in a single lipid. However, the lipid networks in Cytoscape (Figure 5) showed similar trends across lipid classes. Therefore, these changes were further described by the cumulative *p* value, resulting in some groups of lipids showing significant changes. Changes in the lipidome in the mouse brain were more distinct in the male group than in the female group (based on PCA and OPLS-DA). In the brain tissue of the *Folh1* −/– mice, increased levels of PC, PCO, PCP, and PG species (cumulative *p* values 1.0 × 10^–12^, 1.1 × 10^–1^, 1.4 × 10^–1^, and 3.7 × 10^–3^ for −/–♂Y compared to +/+♂Y and <1.0 × 10^–15^, 7.0 × 10^–3^, 1.8 × 10^–4^, and 2.4 × 10^–2^ for −/–♀Y compared to +/+♀Y, respectively) and decreased levels of PE, PEP, PEO, and PI (cumulative *p* values 2.3 × 10^–8^, 2.0 × 10^–5^, 7.5 × 10^–3^, and 5.7 × 10^–2^ for −/–♂Y compared to +/+♂Y and 5.7 × 10^–8^, 8.6 × 10^–5^, 6.1 × 10^–2^, and 2.8 × 10^–1^ for −/–♀Y compared to +/+♀Y, respectively) were detected compared to the *Folh1 +/+* mice. Sphingolipids were significantly decreased in −/–♂Y compared to +/+♂Y, but the change was much less pronounced in −/–♀Y compared to +/+♀Y. Prominent sphingolipids that were decreased were Cer (d18:1/18:1), Cer (d18:1/22:5), HexCer (d18:1/18:0), and HexCer (d18:1/22:1) with *p* values 5.2 × 10^–3^, 1.2 × 10^–2^, 1.4 × 10^–2^, and 6.7 × 10^–2^ in −/–♂Y compared to +/+♂Y, respectively. A decrease in sphingomyelin species with longer acyl chain, such as SM (40:1), SM (42:1), and SM (42:2), was found in both the −/–♂Y (with *p* values 3.7 × 10^–3^, 3.1 × 10^–2^, and 3.8 × 10^–2^, respectively, when compared to +/+♂Y) and −/–♀Y (with *p* values 3.1 × 10^–1^, 3.1 × 10^–2^, ad 1.2 × 10^–2^, respectively, when compared to +/+♀Y) groups. No significant change in polar lipid species, such as LPC, LPCO, LPE, LPEO, and in FA was observed in −/–♀Y. However, in the −/–♂Y group, there was a not significant but clearly visible increase in the LPC, LPCO, and FA species, namely, LPC 18:0, LPC O-16:0, FA 20:1, FA 20:2, and FA 22:4, as compared with +/+♂Y. The increased PC species were mainly highly unsaturated with sum composition, such as 34:4, 36:6, 38:6, 38:7, 41:6, 42:10, and others. The few decreased PC species generally had only one or no double bond. To provide a more in-depth insight into the changes in lipidome occurring in the brain, the molecular composition of lipid species was distinguished on the individual acyl chain level. Acyl chain moieties associated with increased PC species were specific in their high carbon number and highly unsaturated acyl chains, especially the 20:3, 20:4, 22:4, and 22:6 acyl chains in combination with 16:0, 16:1, and 18:1 acyl chains. The analysis of PE, PEP, and PEO lipids, decreased in *Folh1 −/–* mice, revealed acyl-specific composition 20:1 and 20:1p moieties in combination with 18:0, 18:1, 16:0p, 18:0p, and 18:1p chains. Similar trends in these acyl chain variants have been found in multiple lipids (in the aforementioned lipid classes) in the −/–♂Y group, whereas they were less evident in −/–♀Y group.

In the CSF, the overall changes in the lipidome were less apparent than in the brain tissue. However, changes in both the CSF and brain tissue were more pronounced in −/–♂Y (Fgure S3). There was a general decrease in cholesteryl ester species, namely, CE 18:2, 20:4, and 22:6, in both the ♂ and ♀ −/–Y as compared to +/+Y. In the −/–♂Y group, there were an increase in several PCO, PE, and PEP species and a decrease in the LPC species as compared to the +/+♂Y group.

### 2.3 *N*-Acetylaspartylglutamic Acid in Biofluids and Brain Tissue

**Figure 6:**
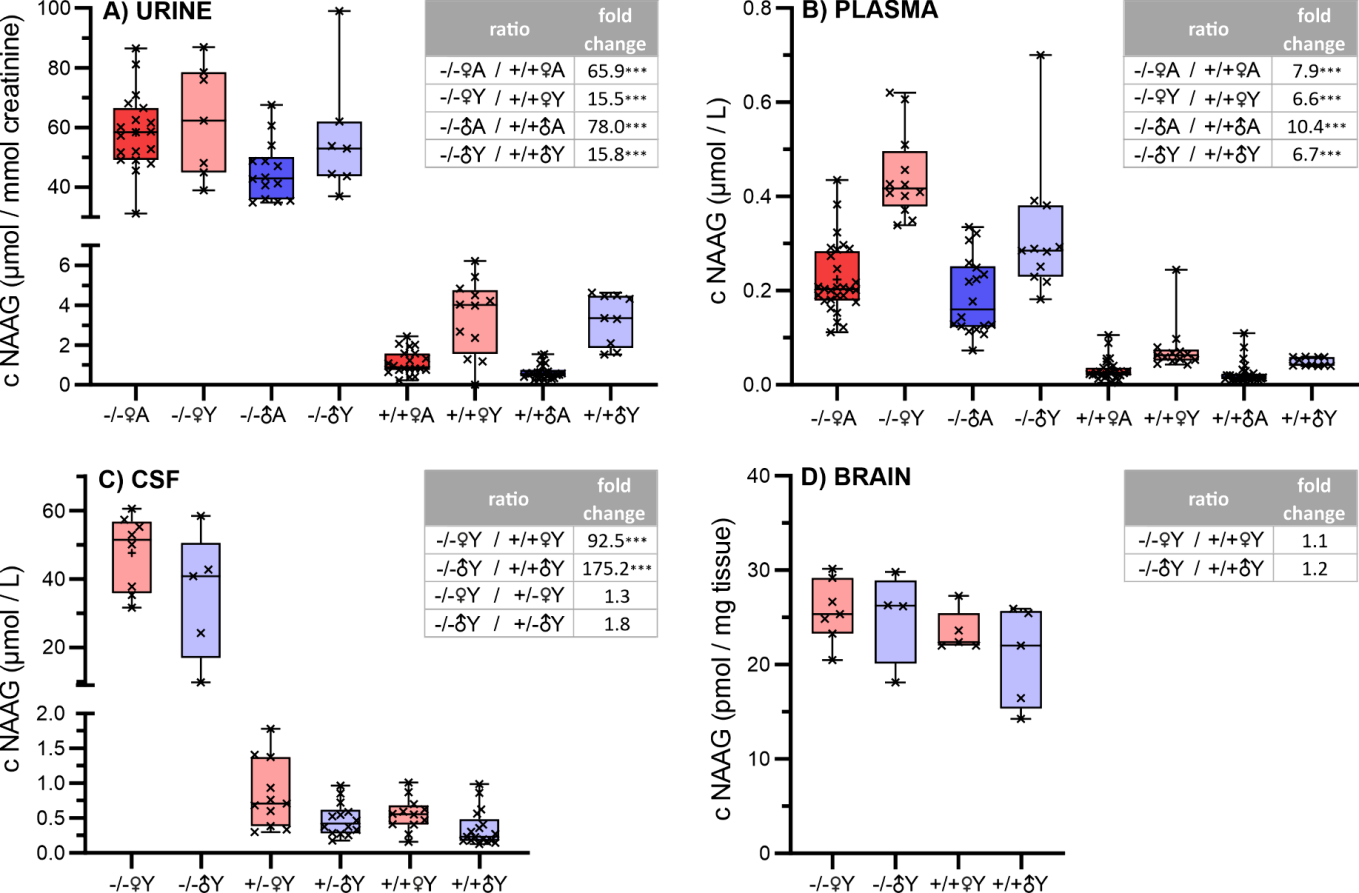
Quantitative analysis of NAAG in the urine, plasma, CSF, and brain from mice of different *Folh1* genotypes. Statistically significant changes are highlighted with asterisks (***, *p* value < 0.001). The boxplot dimensions are equal to the interval between the first and third quartile (interquartile range), the horizontal line corresponds to the median, and whiskers are shown as the minimum and maximum value.

Because NAAG is a known GCPII substrate, we analyzed it separately in all four tested biological materials with a targeted approach. NAAG concentrations in the *Folh1 +/+* mice compared to *Folh1* −/– mice were significantly increased primarily in the urine and CSF (Figure 6). A considerably smaller elevation of NAAG levels was observed in the plasma, and surprisingly, almost no difference in NAAG was found in the brain lysates. Regardless of the genotype observed, NAAG concentrations in plasma and urine were lower in aged mice. Because the largest change (*Folh1* +/+ vs *Folh1* −/−) in NAAG concentration was observed in CSF, the CSF of *Folh1+/–* mice was analyzed to distinguish the effect of deficiency of one GCPII allele. However, no meaningful alteration of the CSF NAAG level in *Folh1+/–* mice were detected.

NAAG not only is a GCPII substrate but also can be degraded by the close homologue of GCPII, glutamate carboxypeptidase III (GCPIII). Thus, we investigated possible changes in NAAG concentrations as well as GCPIII-specific substrate BCG levels in the CSF and brain lysate of GCPIII-deficient mice. Whereas BCG levels in CSF of GCPIII-deficient mice were significantly increased compared to those in wild-type mice, NAAG concentrations were not altered. No meaningful alterations were observed in the brain lysates (Figure S4).

## 3 Discussion

### 3.1 Metabolomic Analysis Revealed Mainly Age- and Sex-Specific Differences

GCPII prevents neuroprotective activity of NAAG by its cleavage^1^ and is highly expressed in prostate cancer,^37^ and hence, it represents a valuable target with diagnostic and therapeutic potentials. GCPII inhibitors, such as 2-(phosphonomethyl)pentanedioic acid (2-PMPA) and *N*-[[[(1S)-1-carboxy-3-methylbutyl]amino]carbonyl]-l-glutamic acid (ZJ43), have shown potential benefits in clinical applications, for example, as neuroprotective agents in ischemic/traumatic brain injury or in amyotrophic lateral sclerosis, as neuromodulators in the treatment of pain or of schizophrenia, or even as radioligands in targeted cancer therapy.^38,39^ Before these new drugs can be introduced into practice, a detailed understanding of biochemical changes in different parts of the body in reaction to these inhibitors is necessary. Because long-term application of GCPII inhibitors could be challenging, the usage of transgenic GCPII-deficient animal models could be beneficial to uncover the consequences of absent GCPII activity. In addition, the characterization of the urinary, plasma, brain, and CSF metabolome in GCPII-deficient mouse models has not been investigated yet.

Both unsupervised and supervised multivariate statistics revealed that the clustering of the groups occurred primarily by age (and mostly by sex) and mainly in urine and plasma, respectively. Although we found a few significant changes in individual metabolites in GCPII-absent mice compared to wild types across all biological materials, these were not systematic, were mainly observed in peripheral biological fluids (plasma and urine), and were not consistent for both sexes. Furthermore, because neither urinary and plasma levels of folate metabolites (dihydrofolic acid, 5-methyltetrahydrofolic acid, and 5-formyltetrahydrofolic acid) nor most other metabolites were significantly altered, we assume that GCPII deficiency has no functional effect on folate absorption or renal function. If physiologically normal changes in the metabolome caused by age and sex serve as a major discriminating factor, it can be inferred that GCPII gene deficiency does not cause significant disruption of the general metabolism. Consistently, previous studies found no significant differences in the phenotype of GCPII-deficient mice.^26,27^ This lack of metabolic changes in GCPII-deficient mice is encouraging for the potential clinical use of GCPII inhibitors where adverse effects need to be closely monitored.

### 3.2 Alterations Occur in the Brain Lipidome of GCPII-Deficient Mice

A detailed description of the lipidome of a GCPII-deficient mouse model has not yet been published to our knowledge. Our findings suggest changes in the myelin of the GCPII-deficient mice based on the decrease of sphingolipid species (SM, HexCer, and Cer), which are generally essential for myelin formation and stability.^40,41^ NAA, the product of NAAG cleavage catalyzed by GCPII, is a main source of acetyl groups for lipid synthesis during brain development.^32^ Disrupted NAA metabolism due to impairment of NAA degradation by aspartoacylase deficiency (Canavan disease) or due to NAA synthesis failure by NAT8L deficiency leads to abnormal myelin composition and reduced sphingomyelin concentration in brain tissue.^31,33^ Therefore, the absence of GCPII activity in astrocytes could be associated with a lack of acetyl groups from NAAG catabolism, resulting in lower levels of sphingolipids. The reduced levels of sphingolipids correlate well with significantly decreased axonal and myelin area of the sciatic nerve in GCPII-deficient mice.^27^ Surprisingly, this axonal and myelin area decline had no adverse physiological effects and even led to faster recovery in sciatic nerve crush tests.^27^ Additionally, we observed a decrease in PE and PE species with alkyl ether (plasmanyl, PEO) and alkenyl ether (plasmalogen, PEP) bonded acyl chains. PEP and PEO are abundant in the brain, especially in the myelin to stabilize and thicken myelin sheets.^42^ Several studies have associated the decrease of PEP in the brain with neurological disorders, such as Alzheimer’s disease,^43,44^ Parkinson’s disease,^45^ or Zellweger syndrome.^46^ The exact mechanism behind the decrease of PEP is not known and is believed to be a combination of oxidative stress, inflammation, peroxisome dysfunction, and remodeling of membrane lipid rafts.^46,47^ PEP species have also been described as potential antioxidants due to correlation with oxidative stress as they deplete from antioxidant pathways, thus serving as free radical scavenger molecules.^48,49^

### 3.3 NAAG and BCG Concentrations in the Biofluids and in the Brain Tissue of GCPII/III-Deficient Mice

Using targeted metabolomics, we found that in GCPII-deficient mice, the most significant changes occurred in the NAAG levels. As compared to +/+♀Y and +/+♂Y, NAAG levels increased 92- and 175-fold in CSF in the −/–♀Y and −/–♂Y groups, respectively. NAAG changes correlate well with known GCPII activity as a NAAG degrading enzyme and with previous results obtained by intraventricular administration of inhibitor ZJ43 in rats. After 1 h administration of the inhibitor, a 512-fold increase of NAAG concentration was observed in CSF with no change in the brain.^50^ However, ZJ43 also inhibits the close homologue of GCPII, glutamatecarboxypeptidase III (GCPIII),^20^ and therefore, it could not be distinguished between effects of inhibitions of these two enzymes.

Given that NAAG acts as a neurotransmitter and is found primarily intracellularly in synaptic vesicles in neurons in the resting state,^51,52^ whereas GCPII is mainly located on the surface or recycling endosomal compartment of astrocytes,^53^ it could be inferred that GCPII mainly regulates NAAG levels in extracellular space. Despite the inconsistency in the measurements among different laboratories, the extracellular basal NAAG concentration using microdialysis method was estimated to approximately 100 nM,^54,55^ whereas the concentration in whole brain lysates was determined as 260 μmol/kg.^56^ This difference is likely due to the clearance of NAAG from the extracellular space by GCPII. Because the major contribution to the total NAAG levels comes from the intracellular environment, this could explain our findings of no statistical difference in NAAG concentrations in the brain lysates between wild-type and GCPII-deficient animals. Moreover, these published concentrations of NAAG in rat CSF bellow 1 μM^56^ agree with our measurements, where the concentration of NAAG in CSF corresponds with that of extracellular fluid. Furthermore, the comparable NAAG levels in the CSF in wild-type and GCPIII-deficient mice suggest that GCPII is the major NAAG-degrading enzyme and that the NAAG increasing effect of inhibitors, like ZJ43, can be linked mainly to GCPII inhibition. In heterozygous animals, which possess approximately half of the NAAG-hydrolyzing activity of the wild-type mice,^24^ NAAG levels are not significantly altered.

The NAAG found in the blood and urine likely originates from the central nervous system,^57^ which could explain the observed higher NAAG concentrations in plasma and urine of GCPII-deficient animals. The highly expressed GCPII on the luminal surface of the proximal tubule of the kidney likely further contributes to a difference in urine that is higher than that in plasma. This is consistent with the characterization of GCPII activity in our GCPII-deficient mouse model,^24,25^ where no compensatory effects were observed and a clear decrease of NAAG cleaving activity was observed with increasing level of GCPII deficiency (*Folh1*^–/–^ ≫ *Folh1*^+/–^ > *Folh1*^+/+^). Despite the white matter diseases linked to NAAG levels like Pelizaeus–Merzbacher disease, Pelizaeus–Merzbacher-like disease, and Canavan disease,^28−30^ a detrimental phenotype was not observed in GCPII-deficient mice;^24^ therefore, the high concentration of NAAG is likely the side effect and not the cause.

### 3.4 Limitations of the Study

Despite the various GCPII-deficient mouse models, there is just one study analyzing how GCPII-deficient mice age,^24^ and it is difficult to follow long-term effects of GCPII absence. Similarly, we investigated the metabolic and lipidomic changes mainly in young mice and only extended it to adult mice using blood plasma and urine samples due to the invasiveness of the CSF/brain tissue sample collection. Because the only known phenotype of aged GCPII-deficient mice is the enlargement of the seminal vesicles,^24^ which could be related to physical activity,^58^ further investigation is still needed. Finally, the use of a methanol extraction, which allows simple parallel metabolomic and lipidomic analysis, makes it impossible to measure highly nonpolar lipids. Another limitation of the study is that we have only studied a limited number of biological materials, and other types of tissues should be investigated in the future. Additionally, even though our targeted methods are high coverage (analysis of over 200 metabolites and 500 lipids), an untargeted metabolomics and lipidomics analysis of absent GCPII mice models would be beneficial for the discovery of changes in unknown molecules. Moreover, because metabolites other than those mentioned above were not analyzed, we cannot completely rule out slight, albeit unlikely, changes in peripheral tissue metabolites that do not affect tissue function.

### 3.5 Conclusions

Using both metabolomic and lipidomic approaches, we found that major changes in the metabolism of GCPII-deficient mice in comparison to the wild-type controls predominantly occurred at the increased NAAG levels. The source of NAAG in neurons and the extracellular location of the active ectodomain of GCPII on astrocytes explain the most pronounced change in NAAG concentration in the extracellular fluid of the central nervous system, herein represented by the concentration in CSF. Secondary changes in plasma and urine are less pronounced. Despite the altered brain lipid composition with reduced levels of sphingolipids and ethanolamine plasmalogens, no detrimental changes in GCPII-deficient mice have been reported so far, probably due to the large compensatory capacity of the brain. Thus, it can be concluded that the short-term absence of GCPII activity does not have a detrimental effect on metabolism (limited to biological materials investigated in this study), which could allow the use of GCPII inhibitors as drug candidates.

## 4 Materials and Methods

### 4.1 Chemicals and Reagents

Acetonitrile (ACN, cat. no. 1000292500), 2-propanol (IPA, cat. no. 1027812500), methanol (MeOH, cat. no. 1037262002) (all LC/MS grade), chloroform (LC grade, stabilized by 0.5–1% ethanol, cat. no. 366927), *tert*-butyl methyl ether (MTBE, cat. no. 650560), ammonium hydroxide (28% aqueous solution, cat. no. 338818), ammonium acetate (AmAc, cat. no. 73594), acetic acid (cat. no. 338826), dimethyl sulfoxide (DMSO, cat. no. 276855) and NAAG (cat. no. A5930) were purchased from Sigma-Aldrich (St. Louis, MO, USA). Deionized water was prepared with a Milli-Q Reference Water Purification System (Molsheim, France). BCG standard was synthesized as described in Návrátil et al. (2016).^5^

### 4.2 Animals

All animal experiments were ethically reviewed, approved by the committee of the Czech Academy of Sciences (No. 92/2020), and carried out in concordance with the European Directive 2010/63/EU. Mice fully deficient in GCPII (C57BL/6N-*Folh1*^em2Ph^/Ph, RRID:IMSR_EM:10058; *Folh1*^–/–^, referred to as −/−), one allele deficient in GCPII (*Folh1*^+/–^, referred to as +/−), and wild type (*Folh1*^+/+^, referred to as +/+) were prepared as described previously^24^ and maintained by cross-breeding to a C57Bl/6NCrl background. The process of preparation of used GCPII-deficient strain and its detailed characterization were described previously.^24^ For simplicity, in the following text, we have introduced the abbreviations +/+, +/–, and −/– for different genotypes (explained further in the text), A and Y for different ages, and male (♂) and female (♀) for different sexes. This description is discussed in more detail in the methods section. Animals older than 60 weeks were considered to be aged, whereas mice younger than 25 weeks were marked as young. Frozen sperm of GCPII-deficient animals is available in the European Mouse Mutant Archive. The GCPIII-deficient mouse model was kindly provided by J.H. Neale, and all measurements were performed after removal of the neomycin cassette and cross-breeding to the C57Bl/6NCrl background (to be published separately). All mice were kept in a pathogen-free environment in individually ventilated cages (maximum six mice per cage) and fed *ad libitum*. This study has an observational design; all animals were included in the study regardless of any extenuating circumstances, and the only randomization applied in this regard was carried out by the experimenter during the random selection of animals that reached the desired age. This study was not preregistered. In addition, no intervention was performed, only a terminal collection of samples from mice of different genotypes, ages, and sexes. To reduce animal suffering, all invasive procedures that had to be performed on live animals were performed under terminal anesthesia using a ketamine (125 mg/kg) and xylazine (20 mg/kg) mixture. This combination of drugs allows for proper anesthesia and pain control without lowering blood and cerebrospinal fluid pressures, which are necessary for sampling. The animals were eventually sacrificed by an overdose of anesthetic or cervical dislocation. The frozen sperm of GCPII or GCPIII-deficient animals will be shared upon reasonable request.

### 4.3 Sample Collection

In total, four biological materials (urine, plasma, CSF, and brain tissue) were used in the study. The study subjects were, according to GCPII genotype, sex, and age, divided into 10 groups: (1) young female homozygotes (−/–♀Y), (2) aged female homozygotes (−/–♀A), (3) young male homozygotes (−/–♂Y), (4) aged male homozygotes (−/–♂A), (5) young female heterozygotes (+/–♀Y), (6) young male heterozygotes (+/–♂Y), (7) young female wild types (+/+♀Y), (8) aged female wild types (+/+♀A), (9) young male wild types (+/+♂Y), and (10) aged male wild types (+/+♂A).

Animals were evaluated randomly after they reached the desired age. Samples were typically taken in the morning unless extenuating circumstances prevented sample collection at that time. The urine was collected after spontaneous urination on a sterile-plastic foil using an automatic pipet right before mice were anesthetized.

Sampling of the CSF was performed as described previously with minor modifications.^59^ Mice were terminally anesthetized with ketamine (125 mg/kg) and xylazine (20 mg/kg) and shaved in the neck area, and the sagittal cut approximately 15 mm long down from the occiput through the skin and superficial layer of muscles was made. The remaining muscles were bluntly dissected and pushed away using microretractors to access the cisterna magna. If bleeding occurred during surgery, then it was stopped by using electrocoagulation. The CSF was collected from the cisterna magna after a puncture of the dura mater using a capillary with a 0.5 mm wide orifice.

The blood was obtained in the terminal ketamine/xylazine anesthesia by cardiac puncture using a heparinized syringe. Subsequently, plasma was separated by centrifugation at 2000*g* for 10 min at 4 °C.

Finally, the brain was collected after euthanasia by cervical dislocation. All samples were stored at −80 °C before processing further.

### 4.4 Sample Preparation

For the metabolomic analysis, 20 μL of plasma or CSF sample was extracted with 80 μL of methanol. The extraction mixtures were vortexed and held overnight at −80 °C. After centrifugation (15,000*g*, 10 min, 4 °C), approximately 70 μL of plasma or CSF supernatant was transferred into a glass vial. Quality control (QC) samples for the relevant kind of matrix were prepared by pooling 5 μL of every plasma or CSF extract sample. Whole brains were mostly used for brain tissue analysis (approximately 400 mg), except for three mice (mouse numbers 57474, 59015, and 59028) where sagittal plane halves (approximately 240 mg) were used because of technical reasons. Samples of the brain tissue were homogenized in 1 mL of cold 80% w/w methanol in water solution using a Tissuelyzer II (Qiagen, Venlo, Netherlands) device (30 Hz, 5 min). The resulting brain tissue lysate (150 μL) was diluted by 80% w/w cold methanol to a final volume of 300 μL and homogenized in a Tissuelyzer II (30 Hz, 3 min) again. The final suspension (200 μL) was then thoroughly centrifuged (21,100*g*, 30 min, 0 °C), and the supernatant was collected, diluted by 80% w/w methanol to the dry weight concentration of either 30 mg/g (whole brains) or 15 mg/g (brain halves) of solution, and stored in −80 °C.

To reduce the variability of the urine samples due to differences in urine osmolality caused by individual differences in a kidneys’ ability to concentrate, all samples were diluted to a final creatinine concentration of 700 μM. Creatinine concentrations in primary samples were determined using a creatinine enzymatic kit (Dialab, Prague, Czechia) according to manufacturer’s protocol with minor modifications that were necessary for assay adaptation to the 96-well plate format. For targeted metabolomic analysis, diluted urine samples were also deproteinized by ultrafiltration using Microcon-10 kDa centrifugal filters (Millipore, Billerica, MA, USA) at 14,000*g* and 4 °C.

Samples for lipidomic analysis of plasma were obtained by mixing 20 μL of plasma with 160 μL of MTBE/MeOH (5:1, v/v). The mixture was shaken for 1 h at laboratory temperature. Subsequently, 40 μL of water was added and allowed to shake for 10 min more. The mixture was then centrifuged (10 min, 14,000*g*, 4 °C), and the top layer was aspirated (100 μL) and then lyophilized. The lyophilized remains were dissolved in 50 μL of IPA/ACN/H_2_O mixture (2:1:1, v/v/v). A 4 μL aliquot from each sample was taken for the QC sample. Lipidomic analysis of the CSF and brain tissue was performed from the methanol extracts prepared for metabolomic analysis, as described above. This was done primarily to reduce the consumption of samples (CSF and brain tissue) that were more difficult to obtain. It should also be noted that the methanolic extract contains all the polar lipid classes, which were of main interest.

### 4.5 Targeted Metabolomic Analysis

Targeted metabolomic analysis of all biological materials was carried out using a liquid chromatography technique connected to tandem mass spectrometry (LC–MS) according to a previously published methodology.^60^ The separation was performed on an UltiMate 3000 Rapid Separation system (Dionex, Sunnyvale, CA, USA), and the data were acquired by a triple–quadrupole mass spectrometer (Triple Quad 6500, Sciex, Foster City, CA, USA). The system was controlled by the Analyst software (version 1.6.2, Sciex, Foster City, CA, USA).

An aminopropyl column (Luna 3 μm NH_2_, 2 × 100 mm, Phenomenex) was used at 35 °C for the chromatographic separation. Mobile phase A consisted of 20 mM AmAc in water (pH 9.75), and ACN was used as mobile phase B. The gradient was set as follows: started with 95% B up to 0.5 min, then linearly decreased to 10% B at 7 min, and stayed at this composition until 13 min. The gradient was then linearly increased back to 95% B at 14 min and stayed at its initial composition until 17 min. Throughout the analysis, the flow rate was 0.3 mL/min.

The ionization was performed in positive and negative modes by polarity switching. The ion source and gas parameters were adjusted: the ion source temperature was 400 °C, the voltage was +5500 and −4500 V, the curtain gas was set to 40 psi, and both ion source gases were set to 40 psi. Scheduled multiple reaction monitoring (MRM) with a window of 2.5 min was applied for the detection of the relevant metabolites. According to previously optimized standards of metabolites, declustering potentials, collision energies, and enter and exit potentials of the collision cell were used. Detailed information and other parameters of the targeted metabolomics method were published in a previous paper.^60^ For the semiquantitative determination of NAAG and BCG, calibration solutions of these standards were prepared in MeOH (for NAAG) and DMSO (for BCG) for optimization of the mass spectrometer parameters. The LC–MS parameters for NAAG were as follows: quantitation and confirmation MRMs (Q1/Q3): *m*/*z* 305.0/148.1 and 305.0/158.1, both in positive ion mode, and a retention time of 7.62 min. The LC–MS parameters for BCG were as follows: quantitation and confirmation MRMs (Q1/Q3): *m*/*z* 320.0/240.0 and 320.0/302.0, both in negative ion mode, and a retention time of 9.26 min. LC conditions were identical to targeted metabolomic analysis. Standard solutions were additionally used for external calibration.

### 4.6 Targeted Lipidomic Analysis

Targeted lipidomic analysis, performed using LC–MS, was adopted from Xuan et al.^61^ The liquid chromatography separation was performed on the ExionLC System (Sciex, Foster City, CA, USA), the data were acquired using a QTRAP 6500+ mass spectrometer (Sciex, Foster City, CA, USA), and the system was controlled by the Analyst software (version 1.6.2, Sciex, Foster City, CA, USA). A reversed-phase BEH C8 column (2.1 mm, 100 mm, 1.7 μm, Waters, Milford, MA, USA) was used for chromatographic separation. Mobile phase A consisted of ACN/H_2_O (3:2, v/v), the mobile phase B was IPA/ACN (9:1, v/v), and both contained 10 mM AmAc. The flow rate was set at 0.35 mL/min, and the column was set at 55 °C. The elution gradient was adjusted accordingly: started with 32% B up to 1.5 min, then linearly increased to 85% B at 15.5 min, then increased again to 97% B at 15.6 min, and kept for 2.4 min. The gradient then reached its initial composition of 32% B at 18.1 min, and it was kept for 1.9 min for column equilibration.

The parameters of the ion source and gases of the mass spectrometer were set as follows: ion spray voltage, +4500 and −4500 V; curtain gas, 40 psi; both ion source gases 1 and 2, 60 and 50 psi, respectively; and source temperature, 400 °C. Data were acquired with scheduled MRM with a window of 2 min. Positive and negative ionization of the compounds in one analysis was performed using the polarity-switching ability of the mass analyzer. Declustering potentials and collision energies for each lipid class were optimized with the SPLASH Lipidomix Mass Spec standard (Avanti Polar Lipids, Alabaster, AL).

The processing workflow included pseudotargeted manual filtering of all of the theoretical MRM transitions based on multiple QC sample measurements. Specific MRM transitions were calculated using the LipidCreator software,^62^ and they were added to the method for identification of lipid molecular species (acyl-specific identification). Correct identification was verified by lipid pattern plots plotted via the R script^63^ and is shown in Figure S1. This verification step was particularly important for correct identification of FA that were measured by the same *m*/*z* value for Q1 and Q3 as they do not yield fragment ions when using standard collision-induced dissociation.

### 4.7 Design of Experiment, Data Treatment, and Statistical Analysis

In planning the experimental design, the power of study and effect size were calculated for all combinations of groups that were taken into statistical analysis. All experiments were double randomized (“RANDARRAY” function in MS Excel) in the steps of sample preparation and run order of analysis. Blinding was not applied for the study. Data from the metabolomic and lipidomic analysis were processed in the SCIEX OS software (Sciex, version 1.6.1) and in the R language (version 4.0.3) using the Metabol package.^64^ The data processing included a QC-based locally estimated smoothing signal correction (LOESS) and a data transformation. Probabilistic quotient normalization with natural logarithm (lnPQN, as described in Dieterle et al.),^65^ Pareto scaling, and mean centering were applied to the final data set. The data from metabolomic and lipidomic analyses, obtained and processed in this form, were considered as relatively quantitative (Table S1). NAAG and BCG concentrations were determined semiquantitatively using external calibration standards. Coefficients of variation (CVs) were calculated from the QC samples, where metabolites or lipids with a CV greater than 30% were excluded from further data processing. Statistical evaluation of the data was performed in GraphPad (version 9.0, San Diego, California, USA), SIMCA software (version 15.0, Umetrics, Umeå, Sweden), and R language (version 4.0.3). The data were evaluated by both multivariate (PCA and OPLS-DA) and univariate (box plots, *t* test, fold change) methods. Additionally, both unsupervised (PCA) and supervised (OPLS-DA) methods were used to prevent potential overfitting in multivariate analysis. For univariate statistical evaluation, the normality of all data sets was tested by the Shapiro–Wilk test (Table S2), where a *p* value > 0.05 together with a passed normality test (a value of “Yes”) and a “not significant, ns” label corresponded to a normal distribution. All data sets provided normal distributions for more than 90% of metabolites/lipids. As most of the data achieved a normal distribution, univariate statistical analysis was performed using a parametric *t* test (two-tailed, unpaired) and calculation of fold-change. The results of univariate statistical analysis for all studied groups are shown in Table S3. The Cytoscape program^66^ was used for global visualization of changes occurring in the brain and CSF lipid profiles. In the Cytoscape visualization (Figure 5; Figures S2 and S3), each of the detected compounds was represented by a circle (node), and significant metabolites/lipids were labeled. The size of nodes was represented by the −log *p* value, and the color span was based on fold-change (FC, shades of red/blue represented an increase/decrease between two tested groups). BF was applied to reduce false positivity of the *t* test. Because metabolites/lipids did not show significant changes after application of BF, *p* value < 0.05 (−log *p* value > 1.3) was used for plotting purposes in Cytoscape.

Differences between cohorts within the entire lipid class were quantified using the cumulative *p* value calculated by Fisher’s method (Table S3). The cumulative *p* value has been used previously to describe cumulative changes in multiple gene expression data sets and can also be used for pathway analysis.^67^ The critical threshold of the cumulative *p* value was adjusted by BF to account for the different numbers of lipids in each lipid class (Table S3).

## Supporting Information Available

Lipid retention pattern plots, overview of changes in the brain and CSF lipidome across all lipid classes as comparison between female (+/+♀Y) and male (+/+♂Y) data sets and comparison of the (−/–♂Y/+/+♂Y) and (−/–♀Y/+/+♀Y), quantitative analysis of NAAG and BCG in the CSF and brain from mice of different NAALAD2 genotypes (PDF)
MS parameters, retention time, and data from metabolomic and lipidomic analysis of plasma, urine, CSF, and brain tissue after processing, normalization, and transformation (XLSX)
Normality testing (Shapiro–Wilk) of the data from metabolomic and lipidomic analysis of plasma, urine, CSF, and brain tissue after processing, normalization, and transformation (XLSX)
Results of univariate statistical analysis (*t* test and fold change) for metabolomic and lipidomic data, calculation of the cumulative *p* value (using Fisher’s method) to evaluate systematic changes in lipid classes in the brain tissue and CSF (XLSX)

## Notes

All animal experiments were ethically reviewed, approved by the committee of the Czech Academy of Sciences (No. 92/2020), and carried out in concordance with European directive 2010/63/EU.

## ABBREVIATIONS

2-PMPA: 2-(phosphonomethyl)pentanedioic acid
ACN: acetonitrile
AmAc: ammonium acetate
BCG: β-citryl-glutamate
BF: Bonferroni correction
CE: cholesterol ester
Cer: ceramide
CSF: cerebrospinal fluid
CV: coefficient of variation
FA: free fatty acid
FC: fold-change
FOLH1: folyl-poly-γ-glutamyl hydrolase I
GCPII: glutamate carboxypeptidase II
GCPIII: glutamate carboxypeptidase III
Hex2Cer: dihexosylceramide
HexCer: hexosylceramide
IPA: 2-propanol
LC–MS: liquid chromatography–tandem mass spectrometry
LPC: lysophosphatidylcholine
LPCO: lysophosphatidylcholine plasmanyl
LPE: lysophosphatidylethanolamine
LPEO: lysophosphatidylethanolamine plasmanyl
MeOH: methanol
mGluR3: metabotropic glutamate receptors
MRM: multiple reaction monitoring
MTBE: *tert*-butyl methyl ether
NAA: *N*-acetyl-aspartyl-aspartate
NAAG: *N*-acetyl-aspartyl-glutamate
NAAGS: *N*-acetylaspartylglutamate synthetase
NAALadase I: *N*-acetylated-alpha-linked acidic dipeptidase I
NAT8L: *N*-acetyltransferase-8-like enzyme
OPLS-DA: orthogonal partial least-squares discriminant analysis
PC: phosphatidylcholine
PCA: principal component analysis
PCO: phosphatidylcholine plasmanyl
PCP: phosphatidylcholine plasmalogen
PE: phosphatidylethanolamine
PEO: phosphatidylethanolamine plasmanyl
PEP: phosphatidylethanolamine plasmalogen
PG: phosphatidylglycerol
PI: phosphatidylinositol
PS: phosphatidylserine
PSMA: prostate specific membrane antigen
QC: quality control
SM: sphingomyelin
