# Supplementary material for: Parallel Metabolomics and Lipidomics of a PSMA/GCPII Deficient Mouse Model Reveal Alteration of NAAG Levels and Brain Lipid Composition
Source: ACS Chem Neurosci. 2024 Feb 20;15(7):1342–55. doi: 10.1021/acschemneuro.3c00494 (PMC10995945; doi:10.1021/acschemneuro.3c00494)
Supplement: Supplementary file 1 — cn3c00494_si_001.pdf [file cn3c00494_si_001.pdf]

Supplementary information

Parallel Metabolomics and Lipidomics of PSMA/GCPII Deficient Mouse Model Reveal Alteration of Brain Lipid Composition

František Sedlák[1,2,3], Aleš Kvasnička[4], Barbora Marešová[1,2], Radana Brumarová[4], Dana Dobešová[4], Kateřina Dostálová[4], Karolína Šrámková[1], Martin Pehr[1,5], Pavel Šácha[1], David Friedecký[4*], Jan Konvalinka[1,6*]

[1] Institute of Organic Chemistry and Biochemistry, Czech Academy of Sciences, Prague 6, Czechia
[2] Institute of Biochemistry and Experimental Oncology, First Faculty of Medicine, Charles University, Prague 2, Czechia
[3] First Department of Internal Medicine - Hematology, Charles University General Hospital in Prague, Czechia
[4]Laboratory for Inherited Metabolic Disorders, Department of Clinical Biochemistry, University Hospital Olomouc, and Faculty of Medicine and Dentistry, Palacký University Olomouc, Czechia
[5]3rd Department of Medicine – Department of Endocrinology and Metabolism of the 1st Faculty of Medicine and General University Hospital in Prague, Charles University, Prague, Czechia
[6]Department of Biochemistry, Faculty of Science, Charles University, Hlavova 8, 128 00 Prague, Czechia

***Corresponding authors:**

David Friedecký, Email: david.friedecky@upol.cz (D.F.), Phone: +420588442619

Jan Konvalinka, Email: konval@uochb.cas.cz (J.K.), Phone: +420220183218;

Figure S1. Lipid patterns plotted separately for each lipid class. Charts are plotted as *m/z* value on the y-axis and retention time in minutes on the x-axis. Saturation equals to the number of double bonds. Lipid pattern plots were used to correctly assign and annotate lipids to annulate potential misidentifications. Few shifts can be observed in a several cases. This was caused by lipid isomers with different acyl-composition on sn-1 and sn-2 positions on the glycerol backbone which differ in retention time, resulting in non-absolute linear/quadratic pattern curve observed (for example in the case of PC 36:4; PC 38:4 and other abundant lipids with multiple isomeric variants). In the case of the LPCO species, the shift is caused by two unresolved types of acyl bonding (ether and vinyl ether).

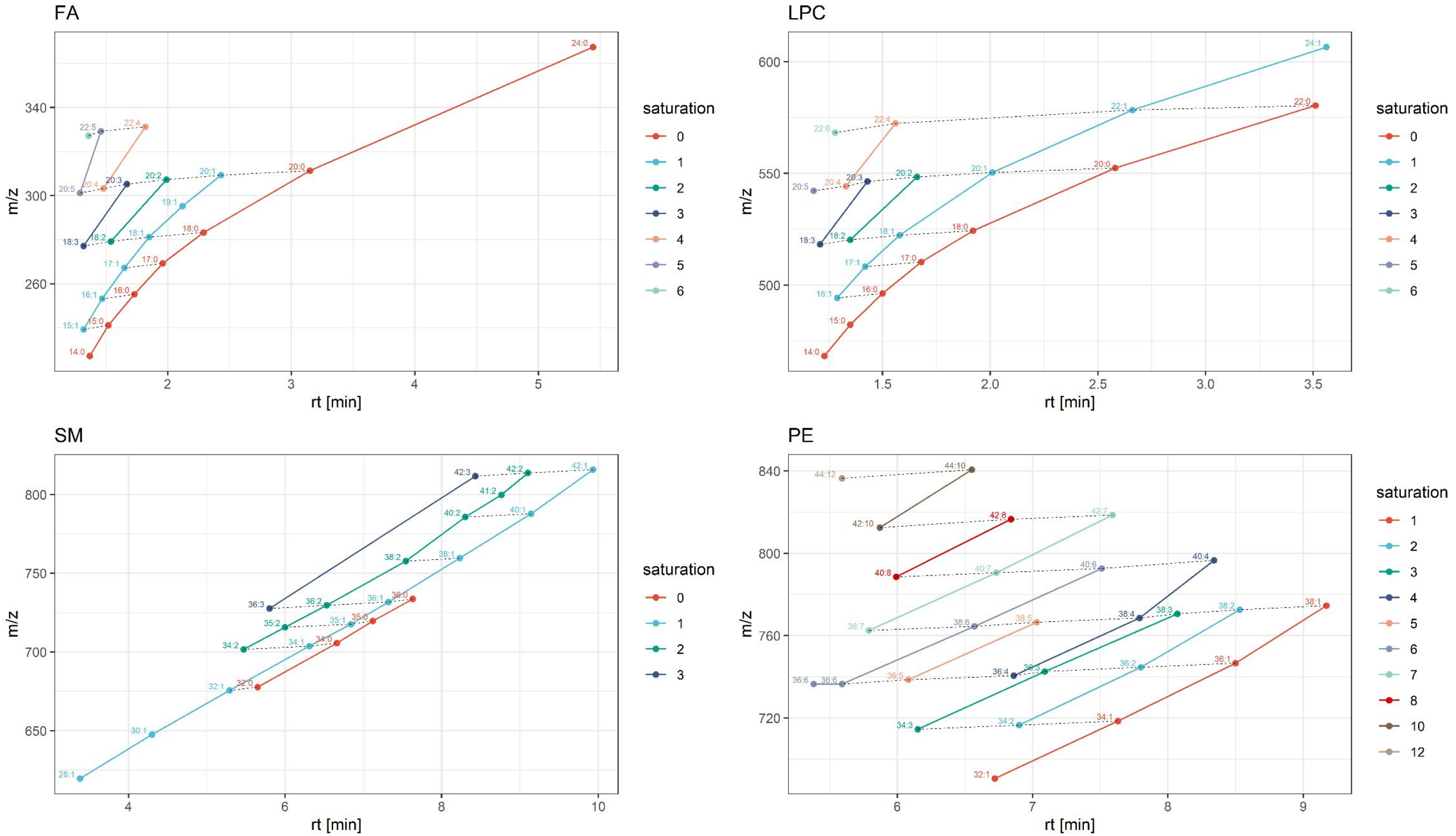

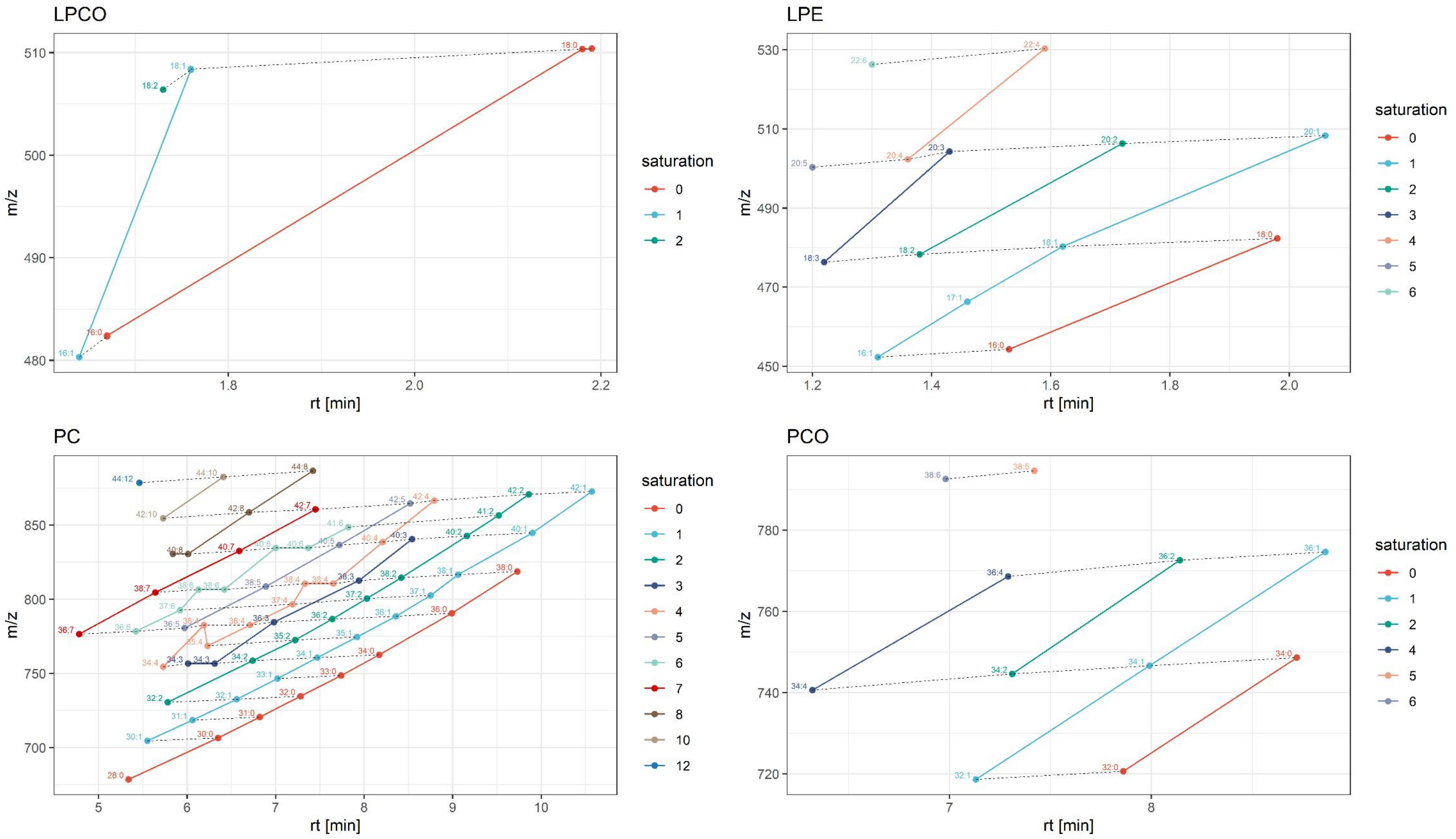
LPCO
m/z
rt [min]
saturation
0
1
2
LPE
m/z
rt [min]
saturation
0
1
2
3
4
5
6
PC
m/z
rt [min]
saturation
0
1
2
3
4
5
6
7
8
10
12
PCO
m/z
rt [min]
saturation
0
1
2
4
5
6

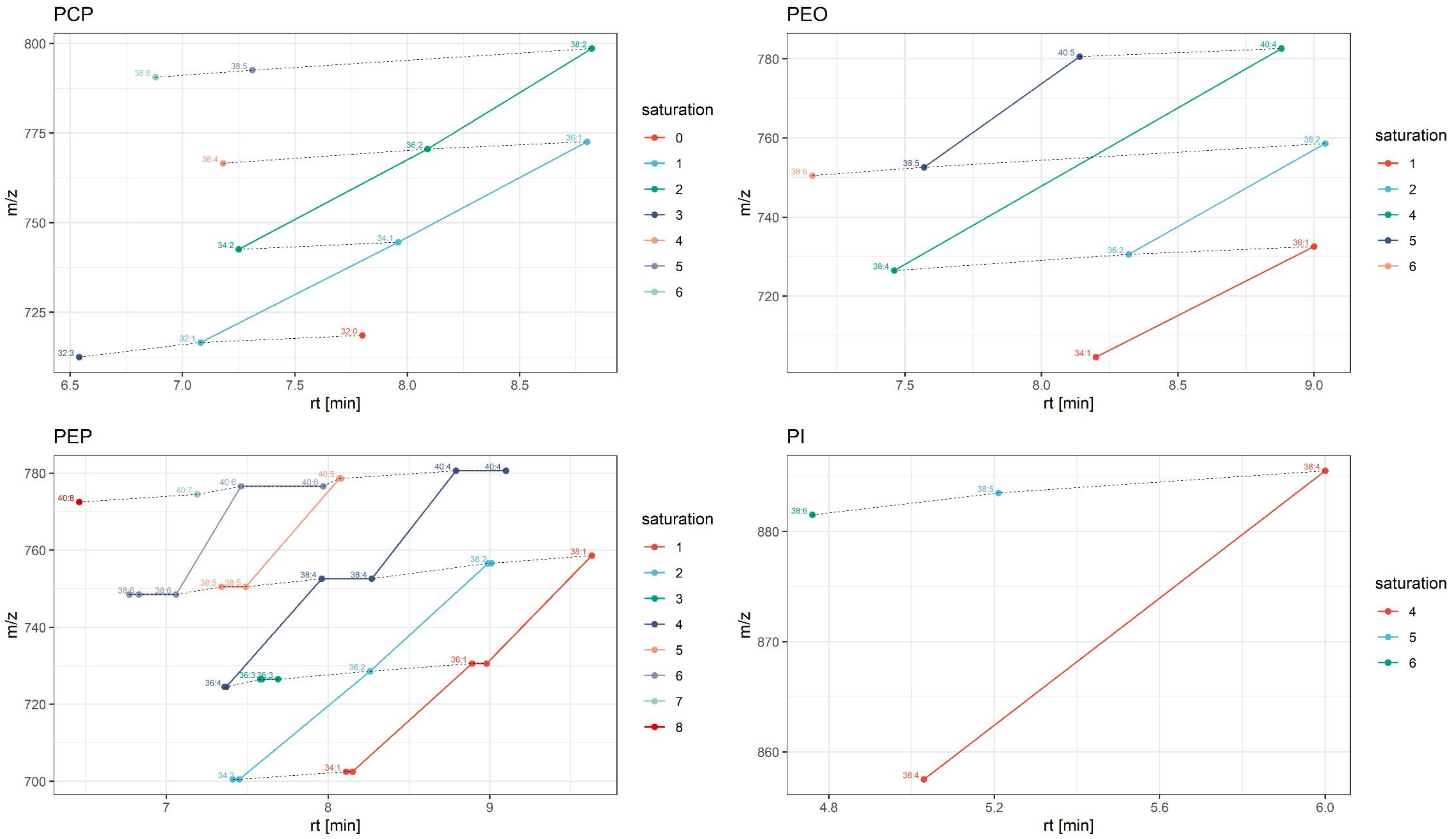
PCP
m/z
rt [min]
saturation
0
1
2
3
4
5
6
38:6
38:5
38:2
36:4
36:2
36:1
34:2
34:1
32:3
32:1
32:0
PEO
m/z
rt [min]
saturation
1
2
4
5
6
40:5
40:4
38:6
38:5
38:2
36:4
36:2
36:1
34:1
PEP
m/z
rt [min]
saturation
1
2
3
4
5
6
7
8
40:8
40:7
40:6
40:5
40:4
38:6
38:5
38:4
38:2
38:1
36:4
36:3
36:2
36:1
34:2
34:1
PI
m/z
rt [min]
saturation
4
5
6
38:6
38:5
38:4
36:4

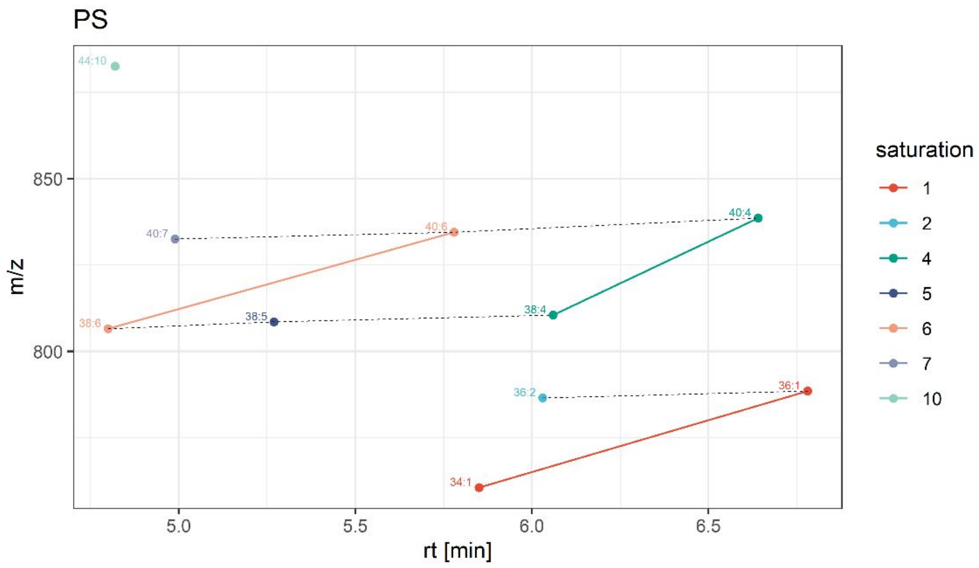
PS
m/z
rt [min]
850
800
5.0
5.5
6.0
6.5
44:10
40:7
40:6
40:4
38:6
38:5
38:4
36:2
36:1
34:1
saturation
1
2
4
5
6
7
10

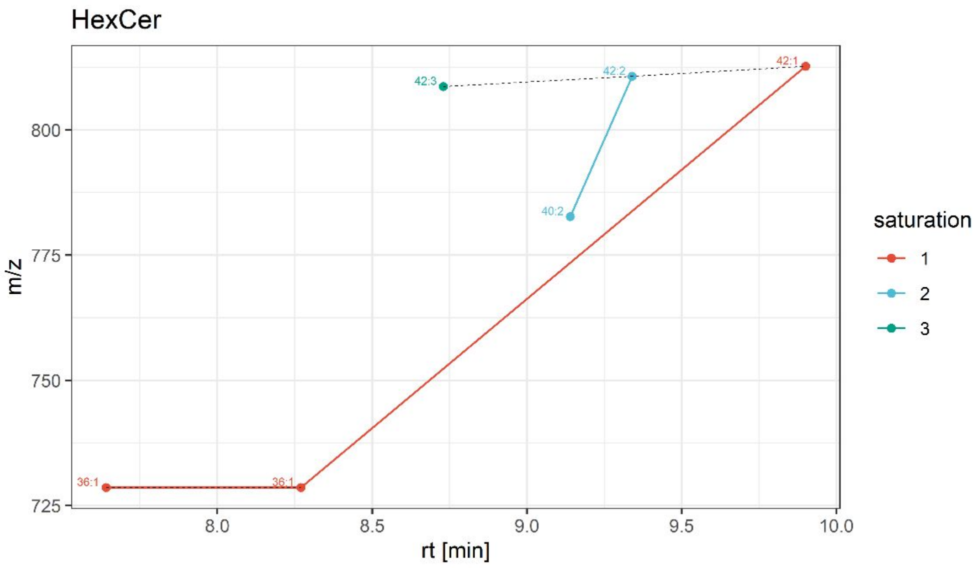
HexCer
m/z
rt [min]
800
775
750
725
8.0
8.5
9.0
9.5
10.0
42:3
42:2
42:1
40:2
36:1
36:1
saturation
1
2
3

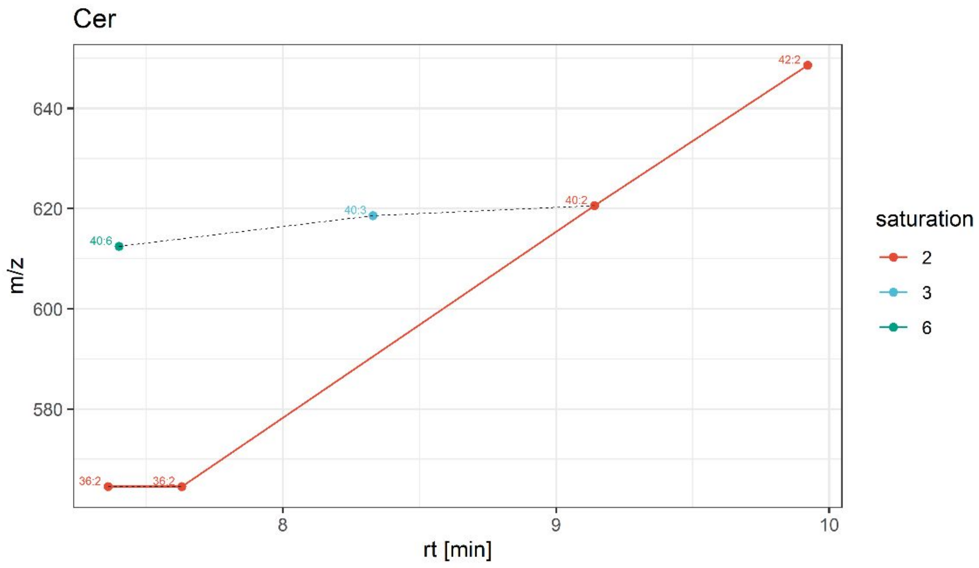
Cer
m/z
rt [min]
640
620
600
580
8
9
10
42:2
40:3
40:2
40:6
36:2
36:2
saturation
2
3
6

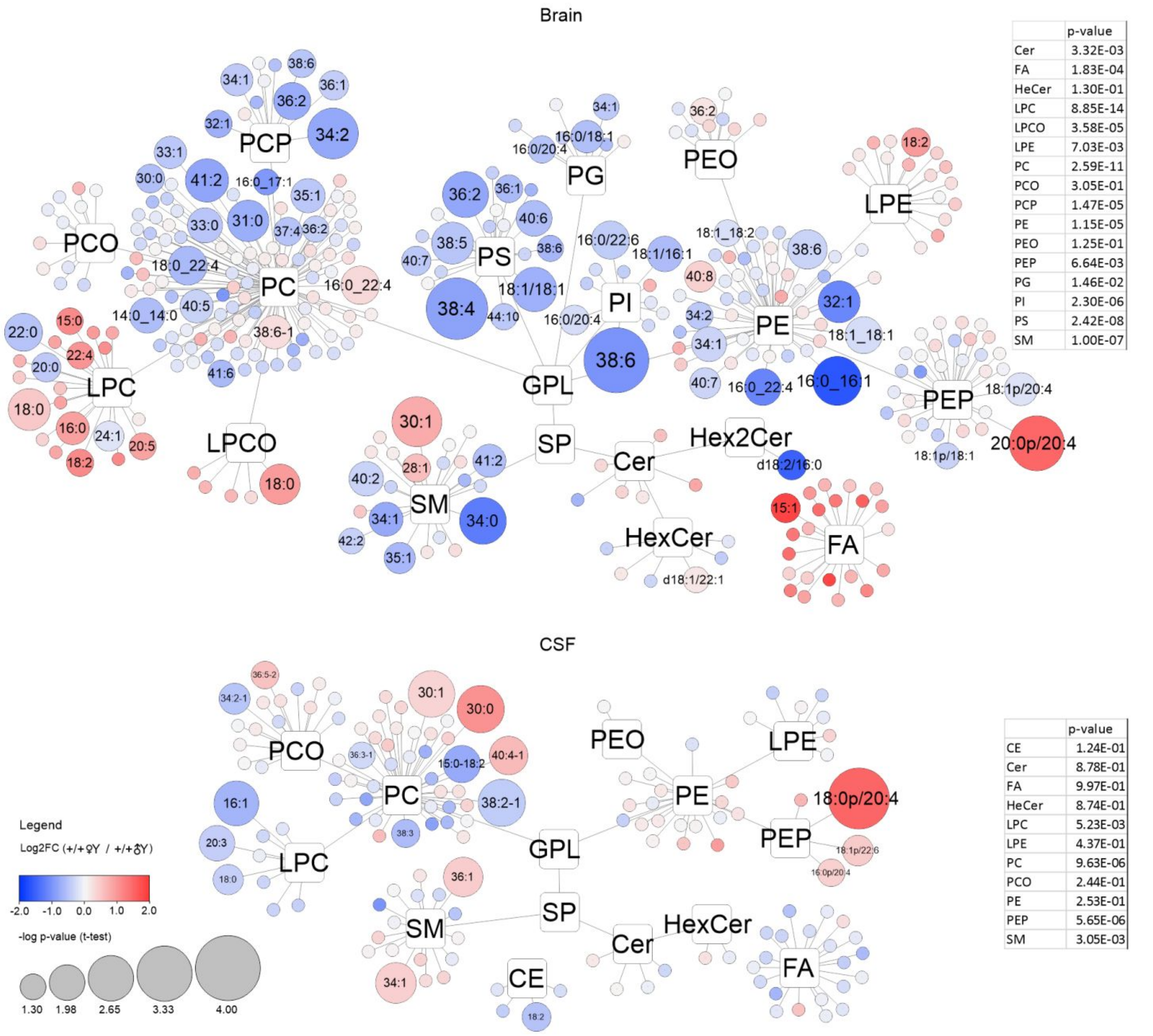

	p-value
Cer	3.32E-03
FA	1.83E-04
HeCer	1.30E-01
LPC	8.85E-14
LPCO	3.58E-05
LPE	7.03E-03
PC	2.59E-11
PCO	3.05E-01
PCP	1.47E-05
PE	1.15E-05
PEO	1.25E-01
PEP	6.64E-03
PG	1.46E-02
PI	2.30E-06
PS	2.42E-08
SM	1.00E-07

	p-value
CE	1.24E-01
Cer	8.78E-01
FA	9.97E-01
HexCer	8.74E-01
LPC	5.23E-03
LPE	4.37E-01
PC	9.63E-06
PCO	2.44E-01
PE	2.53E-01
PEP	5.65E-06
SM	3.05E-03

Figure S2. Overview of changes in brain and CSF lipidome across all lipid classes as comparison between female (+/+♀Y) and male (+/+♂Y) dataset. Color of nodes corresponds to log2FC (blue – decreased and red – increased in the the female group) and their size depends on the p-value (t-test) which is -log transformed for better orientation. Only lipids with a -log p-value greater than 1.3 are shown with labels. Values in the table correspond to a cumulative p-value calculated via the Fisher's method. Labels are shown based on the level of identification confidence as a sum or acyl-chain-specific formula. Cer - ceramides; DG - diacylglycerols; Hex2Cer - dihexosylceramides; HexCer - hexosylceramides; CE - cholesteryl esters; LPE - lysophosphatidylethanolamines; LPG - lysophosphatidylglycerols; LPC - lysophosphatidylcholines; PE - phosphatidylethanolamines; PEP - plasmenyl (plasmalogen) phosphatidylethanolamines; PEO - plasmanyl phosphatidylethnaolamines PG - phosphatidylglycerols; PC - phosphatidylcholines; PCP - plasmenyl (plasmalogen) phosphatidylcholines; PCO - plasmanyl phosphatidylcholines; PI -

phosphatidylinositols; PS - phosphatidylserines; SM - sphingomyelins. The number of biological replicates is described in detail in the Figure 2 in the main text.

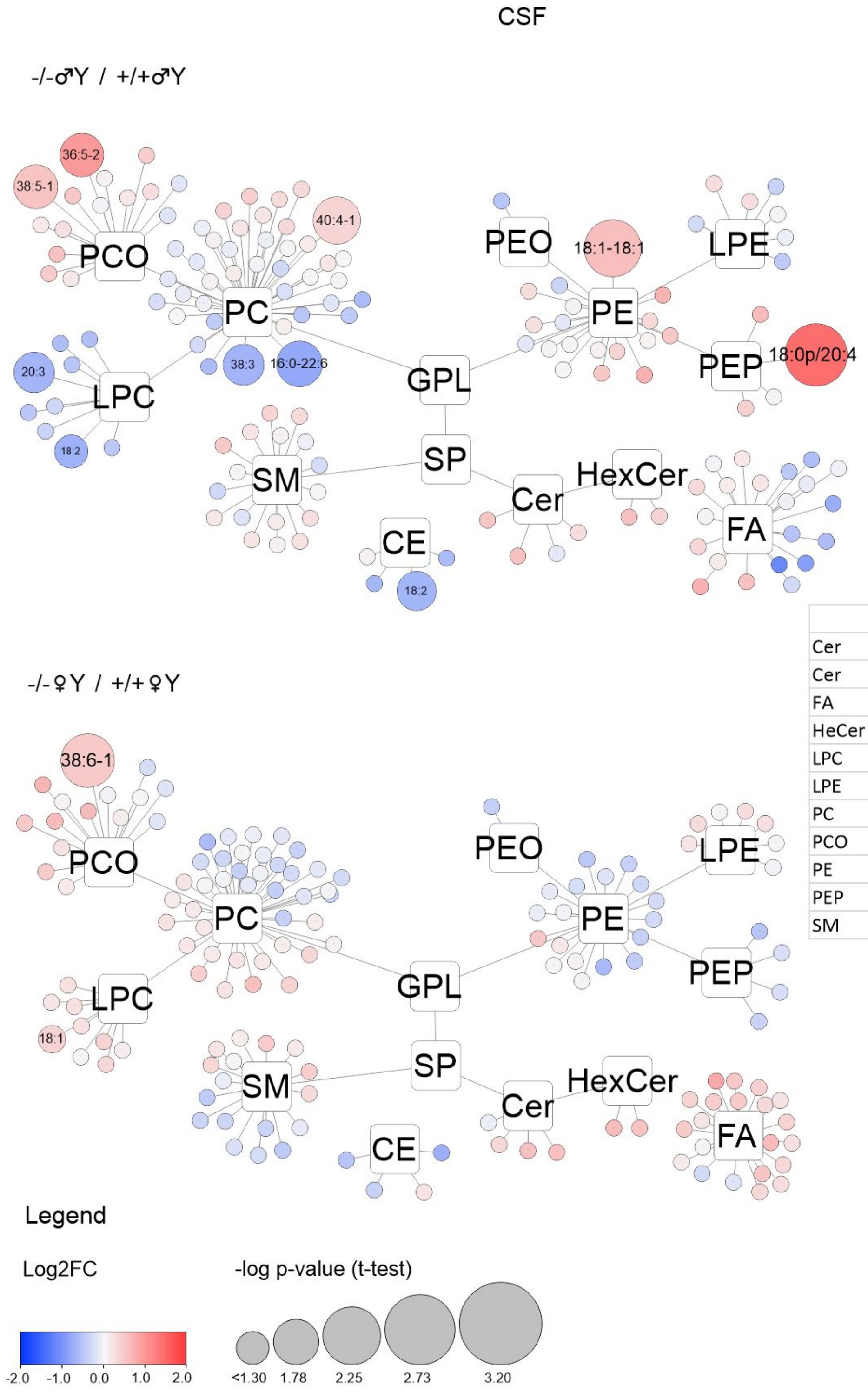

	Males	Females
Cer	9.52E-02	6.66E-01
Cer	2.56E-01	9.89E-01
FA	6.26E-01	1.00E+00
HeCer	6.18E-01	1.00E+00
LPC	4.02E-04	8.16E-01
LPE	4.17E-01	9.97E-01
PC	8.00E-01	9.83E-01
PCO	3.93E-03	5.80E-01
PE	6.46E-01	4.04E-01
PEP	6.11E-02	9.83E-02
SM	9.38E-01	5.41E-01

Figure S3. Overview of changes in CSF lipidome across all lipid classes. Color of nodes corresponds to log2FC (blue – decreased and red – increased in the homozygous group) and their size depends on the p-value (t-test) which is -log transformed for easier orientation. Comparison of the male dataset

displayed on the top (–/–♂Y / +/+♂Y) and female dataset on the bottom (–/–♀Y / +/+♀Y). Only lipids with a -log p-value greater than 1.3 are shown with labels. Values in the table correspond to a cumulative p-value calculated via the Fisher's method. The number of biological replicates is described in detail in the Figure 2 in the main text.

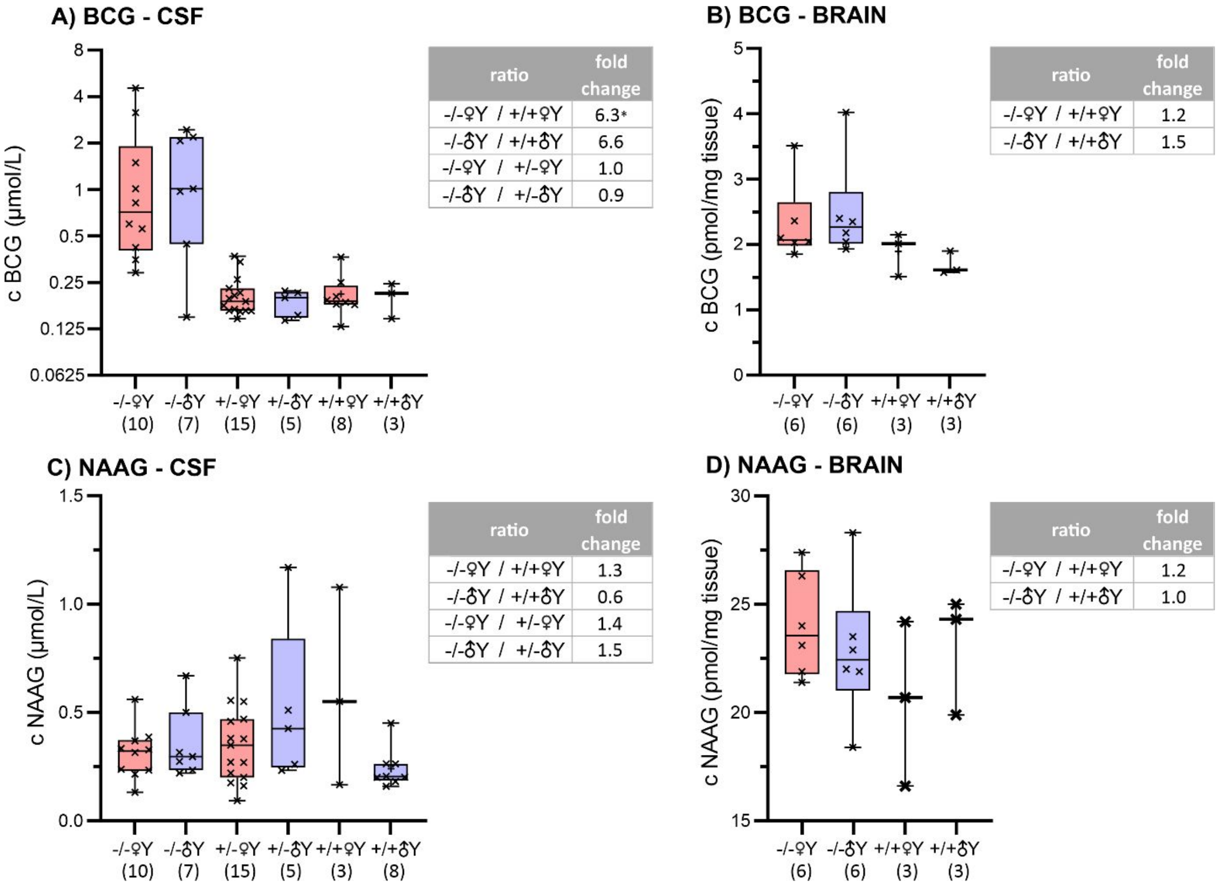

Figure S4: Quantitative analysis of NAAG and BCG in CSF and brain from mice of different *NAALAD2* genotype. Statistically important changes are highlighted with asterisks (*, p-value < 0.05). The numbers under experimental groups correspond to the number of biological replicates. The boxes dimensions equal to the interquartile range, the central horizontal line corresponds to the median, and whiskers are shown as the minimum and maximum value.
